# IL2RG-related immunodeficiencies: from SCID to atypical presentations

**DOI:** 10.3389/fimmu.2026.1703097

**Published:** 2026-03-13

**Authors:** Efrossini Briassouli, Nikolaos Marinakis, Vana Spoulou, Luigi D. Notarangelo

**Affiliations:** 1 Second Department of Paediatrics, National and Kapodistrian University of Athens (NKUA), Aglaia Kyriakou Children’s Hospital, Athens, Greece; 2 Laboratory of Genetics, Department of Medicine, Democritus University of Thrace, Alexandroupolis, Greece; 3 First Department of Paediatrics & Immunobiology & Vaccinology Research Lab, National and Kapodistrian University of Athens, “Agia Sophia” Children’s Hospital of Athens, Athens, Greece; 4 Laboratory of Clinical Immunology and Microbiology, National Institute of Allergy and Infectious Diseases (NIAID), National Institutes of Health (NIH), Bethesda, MD, United States

**Keywords:** atypical X-CID, IL2RG, leaky SCID, maternal T-cell engraftment, somatic reversion, X-SCID

## Abstract

**Background and significance:**

The interleukin-2 receptor gamma chain gene (*IL2RG*) encodes for the common γ chain (γ_c_) protein, that is a shared signaling component of multiple interleukin receptors, including IL-2, IL-4, IL-7, IL-9, IL-15, and IL-21, and plays a pivotal role in lymphocyte development, homeostasis, and function. Mutations in *IL2RG* cause X-linked severe combined immunodeficiency (X-SCID) and a broad spectrum of related phenotypes ranging from typical SCID to leaky or atypical presentations, sometimes mimicking common variable immunodeficiency or immune dysregulation syndromes. Over the last decade (2015–2025), advances in molecular diagnostics, next-generation sequencing, and functional immunology have expanded the known *IL2RG* mutational spectrum and refined genotype–phenotype correlations.

**Recent advances:**

Recent research has uncovered novel hypomorphic variants, revealed the structural basis of receptor dysfunction, and elucidated the impact of specific mutations on JAK–STAT signaling. Longitudinal natural history studies have improved understanding of disease progression in partial loss-of-function cases, while expanded newborn screening for SCID has facilitated earlier diagnosis. Advances in preclinical and clinical gene therapy have addressed historical challenges such as insertional mutagenesis, with emerging protocols achieving stable multilineage immune reconstitution. Moreover, comparative HSCT outcome analyses have informed donor selection, conditioning strategies, and post-transplant care, particularly in resource-limited settings.

**Clinical Impact:**

Improved molecular diagnostics have enabled precision diagnosis in patients with atypical presentations, allowing earlier initiation of curative therapies such as HSCT or gene therapy. Recognition of immune dysregulation, autoimmunity, and malignancy as part of the *IL2RG*-related spectrum has refined long-term follow-up protocols. Multidisciplinary care, integrating infectious disease, immunology, and genetics expertise, has become essential for optimizing patient outcomes.

**Future Directions:**

Ongoing priorities include the expansion of gene therapy trials to cover hypomorphic and late-presenting cases, refinement of reduced-intensity conditioning regimens to minimize toxicity, and development of targeted molecular therapies to modulate downstream signaling in non-transplant candidates. Global initiatives for SCID newborn screening, coupled with collaborative registries, are expected to improve early diagnosis and equitable access to curative interventions.

## Introduction

Pathogenic variants in the *IL2RG* gene – encoding the common gamma chain (γ_c_) shared by multiple interleukin receptors – cause a spectrum of X-linked immunodeficiencies. The classic and most severe phenotype is X-linked severe combined immunodeficiency (X-SCID), characterized by profoundly impaired T-cell and NK-cell development with dysfunctional B cells (1). Affected infants with X-SCID typically succumb to infections in the first year of life if untreated (2).

Over the past years, newborn screening, high-throughput sequencing and curated variant databases have broadened this view, revealing numerous hypomorphic and atypical *IL2RG* variants. These give rise to “leaky” phenotypes and overlapping clinical pictures, including combined immunodeficiency, X-linked lymphoproliferative disease–like syndromes and even common variable immunodeficiency (CVID). Finally, in some cases the clinical and immunological phenotype has been modified by somatic reversion.

In this review, we synthesize current knowledge on IL2RG biology and γc cytokine signaling, and link molecular mechanisms to the evolving clinical spectrum—from classical X-SCID with maternal T-cell engraftment to leaky and CVID-like disease. We summarize genotype–phenotype correlations, diagnostic and functional approaches, and current curative strategies, including hematopoietic stem cell transplantation (HSCT) and gene therapy. Our goal is to provide an up-to-date, clinically oriented reference for immunologists and clinicians involved in the diagnosis and management of IL2RG-related immunodeficiencies.

## *IL2RG* gene and common γ_c_ cytokine signaling

### IL2RG (Common γ_c_) structure and function

The *IL2RG* gene encodes a 369–amino acid type I cytokine receptor subunit (γ_c_, also known as CD132) that is an essential component of at least six interleukin receptors: IL-2, IL-4, IL-7, IL-9, IL-15, and IL-21. The IL2RG protein has an extracellular domain (~23–262 amino-acids) with two fibronectin type III subdomains and conserved motifs (including four cysteines and a WSXWS motif), a transmembrane segment (~263–283aa), and a short cytoplasmic tail (~284–369aa) containing Box 1 and Box 2 regions required for signal transduction (3, 4). IL2RG is broadly expressed on the surface of lymphocytes and their precursors (5). The extracellular portion of γc binds cytokines, while the cytoplasmic tail constitutively associates with the tyrosine kinase JAK3 to transmit signals inside the cell (6). The *IL2RG* gene is located on Xq13.1; accordingly, pathogenic *IL2RG* variants follow X-linked inheritance. Hemizygous males carrying deleterious *IL2RG* mutations develop disease, whereas female carriers are typically immunologically normal due to non-random X-chromosome inactivation favoring the healthy allele (7). Indeed, studies in obligate carrier females have shown that T cells are derived almost exclusively from progenitors expressing the functional *IL2RG* allele (7). This reflects the selective advantage of developing lymphocytes with intact γc signaling in the presence of one mutant allele.

### γc cytokine receptor complexes

The common γ_c_ chain acts as a shared signaling subunit (often described as a ‘‘promiscuous” co-receptor) that pairs with distinct cytokine-specific receptor chains to form heterodimeric or heterotrimeric receptor complexes (8). For example, the IL-7 receptor consists of IL-7Rα paired with γ_c_, IL-21 signals through IL-21Rα paired with γ_c_, and IL-2 and IL-15 utilize a three chain (trimeric) receptor complex that comprises γ_c_, a shared IL-2Rβ/IL-15Rβ chain, and a distinct IL-2Rα or IL-15Rα chain (8). Despite using the same γ_c_ subunit, each cytokine engages its unique receptor and thereby trigger specific biological effects. The affinity of γ_c_ for its partner chains and the exact assembly of the receptor can differ among cytokines, but all γc-family receptors absolutely require the γc chain for high-affinity cytokine binding and signal transduction (7) Importantly, no other protein can substitute for γc in these receptors – which explains why *IL2RG* mutations concurrently disrupt the signaling of multiple cytokines across the immune system.

### JAK-STAT signaling via IL2RG

**Figure 1:**
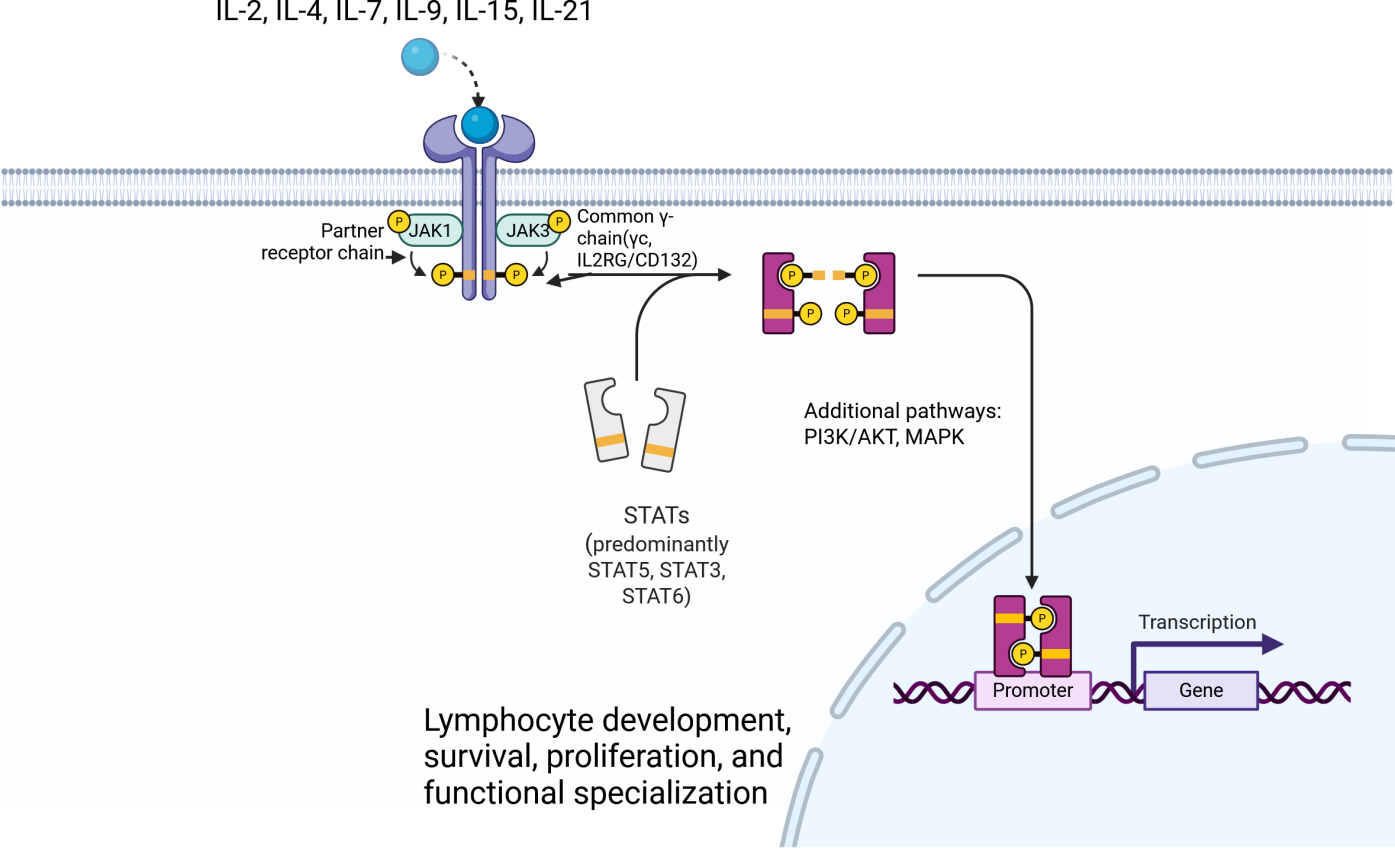
Schematic overview of common γ-chain (IL2RG/CD132)-dependent cytokine receptor signaling. The common γ-chain (γc, IL2RG/CD132) is shared by the receptor complexes for IL-2, IL-4, IL-7, IL-9, IL-15, and IL-21. Cytokine binding promotes receptor assembly with the appropriate partner chains and activation of JAK1 and JAK3, leading predominantly to STAT phosphorylation, dimerization, and nuclear translocation, with additional engagement of PI3K/AKT and MAPK-related pathways. These signaling cascades are essential for lymphocyte development, survival, proliferation, and functional specialization. Created with BioRender.com.

A central function of the IL2RG cytoplasmic tail is to recruit the tyrosine kinase Janus kinase 3 (JAK3). In all γ_c_-containing cytokine receptors, the IL2RG protein constitutively associates with JAK3 via its C-terminal region (which interacts with the FERM domain of JAK3) (9). As a result, bi-allelic loss-of -function mutations in the *JAK3* cause a clinical and immunological phenotype virtually identical to IL2RG deficiency (X-SCID), although JAK3-deficient SCID is inherited in an autosomal recessive (10, 11). When a γ_c_-utilizing cytokine (e.g. IL-2) binds to its receptor complex, the associated JAK3 enzyme is brought into proximity and becomes activated. Specifically, JAK3 (bound to γ_c_) and JAK1 (bound to the corresponding cytokine-specific receptor chain, such as IL-2Rβ for the IL-2/IL-15 receptor) trans-phosphorylate each other, initiating the intracellular signaling cascade (7). The activated JAKs then phosphorylate specific tyrosine residues on the receptor tails, creating docking sites for Signal Transducers and Activators of Transcription (STAT) proteins (1). STAT molecules (particularly STAT5 in many γc signaling pathways) are recruited and phosphorylated, after which they dimerize and translocate into the nucleus to induce transcription of target genes (1). Notably, while all γc-family cytokines primarily activate the JAK1–JAK3–STAT5 pathway, certain cytokines additionally bias signaling toward other STATs: for example, IL-4 strongly activates STAT6, and IL-21 predominantly activates STAT3 (with weaker activation of STAT1 and STAT5) (1). Besides the canonical JAK–STAT route, γc cytokine receptors can also engage secondary signaling pathways such as PI3K/Akt and RAS/MAPK, which promote lymphocyte survival, proliferation, and differentiation (8). The integrated activation of these pathways drives lymphocyte expansion and differentiation in response to cytokines. **Figure 1** provides a schematic overview of the common γc-containing receptor complexes and the downstream JAK–STAT signaling cascade.

### γc Cytokines in Lymphocyte Development and Function

The suite of γ_c_ -dependent cytokines collectively regulates key stages of lymphocyte development, homeostasis, and function (1, 7). IL-7 is indispensable for early T-cell development in the human thymus and for the survival of naive T-cell in the periphery (7). IL-15 is crucial for the development and expansion of natural killer (NK) cells, and it is also required for the long-term maintenance of memory CD8^+^ T cells (12).

Other γc cytokines principally act at later stages of immune responses or in specialized subsets. IL-21, produced mainly by T follicular helper (T_FH_) cells, promotes B-cell differentiation (including class-switch recombination and plasma cell generation) and influences the development of T_FH_ and Th17 T-cells (7). IL-2, while initially identified as a T-cell growth factor, is now recognized as essential for the development of regulatory T cell (Treg) and T- cell peripheral tolerance (7). It also mediates activation-induced cell death of effector T cells, a mechanism that helps terminate immune responses once a pathogen is cleared (13). IL-4 drives the differentiation of CD4^+^ Th2 cells and induces B-cell isotype switching to IgE, while IL-9 supports the growth of mast cells and the differentiation of Th9 cells (both involved in allergic and antiparasitic responses).

Although each cytokine thus has specialized immunological roles, the common γ_c_ chain is the non-redundant linchpin that enables all these signals. Consequently, *Il2rg*-knockout mice or X-SCID patients (who lack a functional γc), exhibit a near-complete arrest of T-cell development (accompanied by an absence of NK cells) due to the combined loss of IL-7, IL-15, and other γc cytokine (1, 7). In particular, the absence of IL-7 is largely responsible for the T-cell developmental block in X-SCID, while the lack of IL-15 accounts for the missing NK-cell population (1, 7). By contrast, B-cell development can proceed in these patients because early B lymphopoiesis does not require γc-dependent cytokines. However, without IL-21 (and the requisite CD4^+^ T-cell help), the patients’ B cells cannot efficiently undergo terminal differentiation into plasmablasts and plasma cells (14). As a result, X-SCID patients develop profound agammaglobulinemia despite having normal or even elevated B-cell counts (1, 15). Thus, IL2RG is absolutely critical for orchestrating normal lymphocyte ontogeny and immune competence across all arms of the adaptive immune system.

## Clinical spectrum of IL2RG-related immunodeficiencies

### Classical X-linked SCID

#### Clinical presentation

Functionally null *IL2RG* mutations cause X-SCID, the most common form SCID in humans. Unless identified at birth through positive newborn screening or a known positive family history of SCID, affected male infants typically present in the first months of life with failure to thrive, chronic diarrhea, and recurrent or life-threatening infections, including opportunistic pathogens such as *Pneumocystis jirovecii* pneumonia, candidiasis, and acute or chronic viral infections due to adenovirus, cytomegalovirus, parainfluenza virus, respiratory syncytial virus and others (2). Skin rash may be observed and is often associated with maternal T-cell engraftment. In its most severe form, this can manifest as generalized erythroderma, mimicking Omenn syndrome. Both severe viral infections and maternal engraftment may induce hyperinflammatory responses with severe liver damage, cytopenias and coagulopathy, closely resembling hemophagocytic lymphohistiocytosis (HLH) (16).

Immunization with live vaccines may lead to serious complications: disseminated or localized BCGosis after immunization with *Bacillus Calmette–Guérin* (17), and profuse, chronic diarrhea after administration of the live oral rotavirus vaccine (18). Physical examination often reveals absent tonsils and lymph nodes due to the lack of lymphocytes. Clinicians must maintain a high index of suspicion for SCID in any infant with unexplained severe or recurrent infections, chronic diarrhea or failure to thrive, especially if lymphopenia or a family history of early infant deaths is noted.

With the implementation of T-cell receptor excision circle (TREC)–based newborn screening in many countries over the last decade, infants with X-SCID are now frequently identified at birth before the onset of infections (15). Similarly, a positive family history for SCID may prompt immunological testing at birth, allowing diagnosis before clinical manifestations appear. Early identification is critical, as untreated X-SCID is invariably fatal in infancy (2). By allowing prompt referral for hematopoietic stem cell transplantation (HSCT) before the development of severe infections, newborn screening has dramatically improved HSCT outcomes for X-SCID, with overall survival exceeding 90% in many series (19).

Typical X-SCID is characterized by profound lymphopenia, especially of T cells: in classical forms, T cells are <50/µL (20), and NK cells are also absent or very low (1). Even in leaky (atypical) forms of X-SCID, the T-cell count at neonatal age is markedly lower than normal (20). In some infants, transplacentally acquired maternal T cells engraft and persist, partially masking the underlying T-cell lymphopenia. These T cells often display an activated, memory phenotype and may cause graft-versus-host disease (GVHD)–like rashes or erythroderma, sometimes mimicking Omenn syndrome. The presence of maternal T cells is considered a pathognomonic sign of profound T-cell immunodeficiency and is discussed in detail in the dedicated subsection on maternal T-cell engraftment (15, 20). The clinical consequences of maternal engraftment are variable: it may cause no specific symptoms or lead to graft-versus-host disease–like manifestations such as rash, hepatosplenomegaly and erythroderma (21).

B cells are present in patients with X-SCID (T^-^B^+^NK^-^ immunophenotype), but they are intrinsically dysfunctional, as they cannot respond to IL-21. Serum immunoglobulins are typically very low (with absent IgA/IgE and variable IgM) due to the lack of T-cell help. Before the introduction of newborn screening, *IL2RG* mutations accounted for approximately 50% of all SCID cases in the United States, but with earlier detection of other genotypes they are now estimated to represent about 30% of cases (22, 23).

#### Molecular features and diagnostic evaluation of classical X-linked SCID

The molecular basis of X-SCID was unraveled in 1993, when independent groups identified mutations in the IL2RG gene in boys with SCID (24, 25). The diagnostic workup of classical X-linked SCID integrates clinical suspicion with a structured immunologic and genetic evaluation. Initial laboratory tests typically show lymphopenia, and lymphocyte subset analysis reveals the characteristic T^-^B^+^NK^-^ immunophenotype, with markedly reduced number of CD3^+^ T cells, absent or very low CD16^+^/CD56^+^ NK cells and preserved or increased number of CD19^+^ B cells. Maternal T-cell engraftment may confound interpretation of T-cell counts and phenotype and should always be considered in IL2RG-deficient infants with unexpectedly preserved T-cell numbers (see ‘Maternal T-cell Engraftment in IL2RG-Related SCID’.

In patients with typical X-SCID, serum immunoglobulins are low, with absent IgA and IgE and variable IgM, and vaccine-specific antibody responses are impaired. T-cell proliferation to mitogens such as phytohemagglutinin is severely reduced or absent. Newborn screening by quantification of T-cell receptor excision circles (TRECs) on dried blood spots typically demonstrates very low or undetectable TRECs. In IL2RG-deficient patients, thymic tissue is severely atrophic, with lack of corticomedullary demarcation and near-complete absence of thymocytes (26). Flow cytometric staining for CD132 (γc) can reveal absent or reduced expression on lymphocytes in classical IL2RG-deficient SCID (27), whereas JAK3 protein expression is preserved, helping to distinguish IL2RG from JAK3 deficiency. Functional assays further support the diagnosis: phospho-flow cytometry demonstrates absent or severely impaired STAT5 phosphorylation in response to γc-dependent cytokines (e.g. IL-2, IL-7, IL-15), with preserved responses to non–γc cytokines. In addition, stimulation of patients’ B cells with CD40 ligand (CD40LG) and IL-21 (mimicking T follicular helper cell signals) fails to induce plasmablast differentiation, reflecting the inability of B cells to respond to IL-21 (14, 29). Finally, targeted genetic testing confirms a hemizygous pathogenic *IL2RG* variant. Over 140 unique (possibly) disease-causing IL2RG variants have been reported (30), scattered throughout the 8 exons of the gene. The majority are loss-of-function changes that result in truncated or absent γc protein and therefore cause a classical SCID phenotype (4). If *IL2RG* sequencing is normal or reveals only variants of uncertain significance in a patient with a T^-^B^+^NK^-^ phenotype, JAK3 sequencing and/or a comprehensive SCID gene panel are indicated. Studies for maternal T-cell engraftment (HLA typing or short tandem repeat analysis) should be incorporated when T-cell numbers appear discordant with the clinical and functional picture.

#### Pathophysiology of immune dysfunction

The immune failure in IL2RG deficiency stems from the inability of lymphoid progenitors to receive key cytokine signals required for development and homeostasis. In the thymus, IL-7 signaling is absolutely required for T-cell generation; IL2RG-null progenitors cannot progress beyond the early T-cell stages, resulting in a SCID phenotype (7), as also confirmed by autosomal recessive IL-7R deficiency (31). Similarly, IL-15–mediated signaling in bone marrow and peripheral niches is required for NK-cell differentiation; in X-SCID, NK cells are therefore profoundly reduced (1).

B lymphocytes do develop (driven by IL-7–independent pathways in the bone marrow), but they remain unable to undergo immunoglobulin heavy-chain class switching or to effectively differentiate into memory B cells or plasma cells in response to IL-21 (and, to some extent, IL-4) (14, 29). Moreover, the lack of T cells results in the absence of T helper signals for B cells. As a consequence, B cells in X-SCID are present but non-functional, and immunoglobulin replacement therapy is required until definitive treatment is achieved (15).

IL-2–mediated signaling is also needed for thymic regulatory T-cell (Treg) development; thus, X-SCID patients lack Tregs, which contributes to immune dysregulation and, when some T cells are present (e.g. maternal cells or in hypomorphic “leaky” forms), may predispose to autoinflammatory complications such as Omenn-like features or cytopenias (7, 15). In typical forms of X-SCID, however, autologous T cells are absent, so autoimmunity is not a prominent feature; instead, extreme vulnerability to all types of infection is the defining clinical hallmark. X-SCID has also been associated with an increased risk of malignancies (e.g. EBV-driven B-cell lymphomas) if immunity is not restored, due to the lack of effective immune surveillance (32, 33). In summary, IL2RG mutation causes a collapse of adaptive immunity and NK-mediated innate immunity, explaining the extreme clinical severity of this disease. In a substantial subset of infants with X-SCID, the intrinsic T-cell lymphopenia may be partially masked by transplacentally acquired maternal T lymphocytes. These engrafted cells can transiently normalize T-cell counts yet fail to provide effective protective immunity and, in some cases, drive graft-versus-host disease–like manifestations. Because maternal T-cell engraftment has important diagnostic and therapeutic implications, it warrants separate consideration.

### Maternal T-cell Engraftment in IL2RG-Related SCID

Transplacental engraftment of maternal T lymphocytes is a well-recognized phenomenon in infants with severe combined immunodeficiency (SCID), including X-SCID. In the absence of functional host T cells, maternal T cells that cross the placenta are not rejected and can persist in the newborn’s circulation. Large series have documented maternal T-cell engraftment in approximately one quarter to one third of SCID patients, highlighting its clinical relevance (15, 16).

Clinically, engrafted maternal T cells may be asymptomatic or may cause a spectrum of graft-versus-host disease (GVHD)–like manifestations. Affected infants can present with erythematous or exfoliative rash, alopecia, hepatosplenomegaly, lymphadenopathy, diarrhea, liver dysfunction and cytopenias, sometimes overlapping with Omenn syndrome (17, 18). In other cases, maternal engraftment is discovered incidentally during immunologic workup or at the time of hematopoietic stem cell transplantation (HSCT). Importantly, despite their presence, maternal T cells do not restore protective immunity, and SCID infants with engraftment remain at high risk for severe infections and early death if untreated (17).

Immunologically, maternal engraftment can partially “normalize” or even increase the absolute T-cell count, masking the underlying T-cell lymphopenia. Engrafted cells usually display a mature, memory phenotype (CD45RO^+^, often HLA-DR^+^), with a restricted T-cell receptor (TCR) repertoire and limited or abnormal proliferative responses to mitogens (18–20). Phenotypically, they characteristically differ from the naïve CD45RA^+^ recent thymic emigrants expected in healthy neonates. Chimerism studies (HLA typing, short tandem repeat [STR] analysis or other methods) confirm the maternal origin of these cells and are critical when the T-cell count appears discordant with the severity of the immunodeficiency (21, 22).

Distinguishing maternal T-cell engraftment from leaky SCID or Omenn syndrome is essential, as the prognostic and therapeutic implications differ. Diagnostic clues include: (i) a memory-skewed T-cell phenotype in a neonate with suspected SCID, (ii) evidence of mixed lymphocyte chimerism with non-infant HLA alleles, and (iii) oligoclonal or restricted TCR usage (15, 18). Recognition of maternal engraftment confirms the severity and chronicity of the underlying immunodeficiency, prevents misinterpretation of T-cell counts, and has practical implications for HSCT planning, including conditioning strategies and donor selection (23, 24).

### Atypical IL2RG deficiencies: hypomorphic and late-onset phenotypes

While *IL2RG* null mutations cause a typical infant-onset SCID phenotype, a growing number of hypomorphic *IL2RG* variants have been reported that permit partial γc expression and signaling. These mutations produce “leaky” phenotypes with some T and/or NK cells present, often allowing survival beyond infancy without immediate transplantation. Over the last decade, such cases have broadened the clinical spectrum of IL2RG-related immunodeficiency to include combined immunodeficiency (CID), X-linked lymphoproliferative (XLP)–like disease, and common variable immunodeficiency (CVID)-like presentations. A 2019 review identified 39 patients with hypomorphic *IL2RG* variants and heterogeneous phenotypes, ranging from early-onset infections to adult-onset disease (4). These variants represent approximately 10% of all *IL2RG* mutations in curated databases (4). Since then, additional cases have been published, including leaky SCID/X-CID, late-onset CID and CVID-like presentations, so that more than 50 atypical IL2RG patients are now described worldwide (3, 25–28).

There is significant heterogeneity of the clinical and immunological phenotype among patients with hypomorphic *IL2RG* mutations. In the 2019 review, age at presentation ranged from infancy to adulthood (4). Some patients had opportunistic infections early in life, while others presented later with chronic lung disease, bronchiectasis or EBV-related complications. Most had reduced but not absent T cells and near-normal NK-cell counts, in contrast to the classic T^-^B^+^NK^-^ phenotype of null *IL2RG* mutations (4). Finally, a subset of these hypomorphic *IL2RG* variants underlies what has been termed atypical X-linked combined immunodeficiency (X-CID), in which T cells are reduced but not absent and NK cells are often present (T^low/normalB^+ NK^+/low). In such patients, T cells often are oligoclonal and show an activated phenotype.

Even within the same genotype, phenotypes can diverge. The missense mutation p.Arg222Cys is a well-characterized “leaky” IL2RG variant reported in at least 18 patients. Some carriers presented with typical SCID in infancy, whereas others had atypical phenotypes and survived longer without early transplant (3, 42). In one analysis, 10 Arg222Cys cases were classified as atypical SCID, while 8 had classic SCID presentations (3). This intra-genotypic variability suggests that modifier genes and environmental factors (including infectious exposures) modulate disease severity.

Intercurrent infections frequently act as triggers that unmask an underlying leaky SCID. Many patients with hypomorphic *IL2RG* variants remain relatively well until a severe infection or live vaccine precipitates a crisis—for example, severe varicella at age 2 years led to the diagnosis of X-SCID in one patient previously thought to have nonspecific immunodeficiency. In the absence of such triggers, patients may be managed for years as “CID” or “CVID” with immunoglobulin replacement and antibiotics, only to deteriorate abruptly with life-threatening infections or HLH.

Recognition of this continuum of IL2RG-related disease is crucial: *IL2RG* mutations do not invariably equate to infant-onset SCID. Instead, there is a spectrum from classic SCID to partial immunodeficiency with autoimmunity, EBV-driven HLH, hyper-IgE dermatitis and CVID-like disease (28). Awareness of these atypical presentations, combined with targeted genetic and functional testing, allows timely consideration of definitive therapy (HSCT or gene therapy) even in older children and adults.

### Omenn-like disease

A subset of patients with hypomorphic *IL2RG* mutations or *IL2RG* reversion somatic mosaicism present with Omenn-like disease. They show generalized erythroderma, lymphadenopathy, hepatosplenomegaly, eosinophilia, markedly elevated IgE and oligoclonal, activated memory-phenotype T cells infiltrating skin and gut, closely resembling “classical” Omenn syndrome described in other leaky SCID genotypes (29). In IL2RG deficiency, residual γc expression supports limited thymic output of autoreactive T cells, while the profound deficiency of regulatory T cells and B-cell help facilitates uncontrolled Th2-skewed inflammation. Representative examples include the splice-site *IL2RG* mutation with revertant T-cell mosaicism reported by Wada et al. (30) and more recent hypomorphic alleles with partial STAT5 signaling defects (3, 4).

From a diagnostic perspective, Omenn-like IL2RG deficiency should be suspected in boys with early-onset eczema or erythroderma, recurrent infections and failure to thrive, even if absolute lymphocyte counts are in the low-normal range. Key immunologic clues include reduced naïve CD4^+^ T cells with skewed or oligoclonal TCR Vβ repertoire, impaired proliferation to mitogens, dysgammaglobulinemia and defective IL-2/IL-7–induced STAT5 phosphorylation on phospho-flow. Maternal T-cell engraftment must be carefully excluded, and prompt genetic testing for IL2RG is essential, as these patients benefit from early HSCT or gene therapy but are otherwise at risk of being misdiagnosed as having isolated atopic or autoimmune disease.

### EBV-driven HLH and XLP-like presentations

A particularly important subgroup of patients with hypomorphic *IL2RG* mutations present with severe Epstein–Barr virus (EBV) infection and hemophagocytic lymphohistiocytosis (HLH), mimicking X-linked lymphoproliferative disease (XLP). In these patients, some T and NK cells are present but functionally impaired and fail to control EBV-infected B cells.

Cases have been reported in which individuals with hypomorphic *IL2RG* variants developed fulminant infectious mononucleosis and HLH upon primary EBV infection (16). In one series, 5 of 6 infants with SCID who developed HLH were found to have *IL2RG* mutations (16). The underlying mechanism likely involves defective CD8^+^ cytotoxic T-cell and NK-cell responses, due to impaired γc cytokine signaling (IL-2, IL-15 and IL-21), resulting in uncontrolled EBV proliferation and hyperinflammation.

Clinically, these patients may initially be suspected to have classical XLP (due to *SH2D1A* or *XIAP* mutations) until genetic testing reveals an *IL2RG* variant. The term “XLP-like” has therefore been applied to some hypomorphic X-SCID cases. EBV-driven HLH in an infant or young child – particularly when accompanied by other atypical or recurrent infections – should prompt evaluation for SCID (16). Importantly, in IL2RG-related disease, HLH reflects an underlying severe immunodeficiency that requires definitive immune reconstitution (HSCT or gene therapy), rather than HLH-directed therapy alone.

In other cases of leaky *IL2RG* defects, EBV can drive lymphoproliferative disorders with expansion of γδ T cells, sometimes manifesting in adolescence or adulthood (35). Additionally, chronic active EBV infection and EBV-positive B-cell lymphoproliferation have been described in older IL2RG-deficient patients who escaped early diagnosis (33). Beyond EBV, other viruses such as adenovirus, CMV and varicella can similarly trigger HLH in partially immunodeficient patients. Together, these observations show that hypomorphic *IL2RG* variants can phenocopy XLP, predisposing to HLH and lymphoma in the context of defective γc signaling.

### CVID-like presentations and somatic reversion

Several male patients with hypomorphic *IL2RG* variants have presented in adolescence or adulthood with recurrent bacterial infections and hypogammaglobulinemia, meeting clinical criteria for CVID. However, the underlying defect is impaired γc expression and/or signaling.

One such patient carried an *IL2RG* promoter mutation (c.-116C>T) in an Ets-binding site, which reduced *IL2RG* transcription and led to low NK-cell numbers and B-cell dysfunction; he was diagnosed with CVID based on recurrent infections and low immunoglobulin levels (36). Another report described three male cousins with the Asp134Val IL2RG variant, who presented in childhood or adolescence with recurrent bacterial infections and absent antibody responses to vaccines; one cousin also had recurrent varicella and sclerosing cholangitis due to *Cryptosporidium* infection (37). In other families, splice-site variants in *IL2RG* generated multiple transcripts, some permitting residual γc expression and function; these were associated with poorly functioning T cells, normal NK-cell counts and normal immunoglobulin levels, but increased susceptibility to infections (38).

In the NIH cohort, a male patient diagnosed with CVID at 5 years of age developed granulomatous skin lesions with non-clonal T-cell infiltrates. At age 27, he had normal total T-cell numbers but low CD4^+^ counts; B cells were present but failed to undergo isotype switching. Genetic testing revealed somatic mosaicism for an IL2RG missense mutation (p.Leu87Pro), with a predominance of mutated alleles and a minor wild-type population; all T cells and a subset of B cells expressed γc, consistent with a reversion event in a committed lymphoid progenitor (39). Another man, initially diagnosed with CVID in childhood, was later found to have somatic mosaicism for an *IL2RG* mutation at age 23, with a mixture of normal and mutant cells due to somatic reversion (39). Emergence of revertant B-cell clones carrying a wild-type *IL2RG* sequence enabled partial antibody production and a milder clinical course (39). These cases illustrate how IL2RG-related disease can masquerade as CVID when somatic reversions partially restore function.

### Somatic reversion

Overall, revertant mosaicism is a recurrent phenomenon in IL2RG deficiency. Spontaneous back-mutations or second-site compensatory changes in the *IL2RG* gene can restore γc expression and signaling in some progenitors, which then repopulate the T- and/or B-cell compartments and partially correct the immunodeficiency (39–41). Somatic reversion events include true back-mutations (restoring the original sequence) and second-site compensatory mutations in the *IL2RG* gene. In some cases, the presence of revertant T or B cell populations can dramatically improve immune function; however, predicting the clinical effects of somatic reversions is not simple, as they are determined by the cell lineage in which the event occurred (hematopoietic progenitor cell versus a progenitor already committed to the T, B, or NK cell lineage) and, in the case of second-site revertants, by the capacity of the revertant to fully or partially restore γ_c_ expression and function. Reversion events have been particularly noted in *IL2RG* missense mutations like p.Arg222Cys (3), perhaps because even slight improvements in γ_c_ folding/conformation give a growth advantage to lymphocyte clones. This phenomenon complicates genotype–phenotype correlation, since a patient’s clinical phenotype may be “rescued” by mosaic reversion even for a severe genotype. It also underscores that some hypomorphic mutations might have been lethal if not for reversion.

Clinically, revertant mosaicism should be suspected if a patient with an *IL2RG* mutation has more T cells than expected or a surprisingly mild course, and especially if he is diagnosed later in childhood or even at adult age. Patients with revertant *IL2RG* mutations often experience milder infections and may be misclassified as having less severe PID (e.g. CVID) until careful analysis reveals an underlying X-linked IL2RG defect. Clues favoring IL2RG-related disease over idiopathic CVID include: childhood onset of symptoms (often since infancy), evidence of T-cell lymphopenia or dysfunction (whereas typical CVID is primarily B-cell–driven), and an X-linked pattern in the family. The identification of revertant *IL2RG* cases also provided proof-of-principle for gene therapy, as naturally occurring corrections demonstrated that restoration of γc function in a fraction of lymphoid progenitors can confer sustained clinical benefit (40, 41). **Tables 1** summarizes the reported cases of CVID-like and somatic reversion presentations in patients with IL2RG mutations, including immunologic features, mutations, and clinical course.

**Table 1:** Published cases of CVID-like and IL2RG reversion immunodeficiency.

Case (Age at Onset – Diagnosis)	IL2RG Mutation (Inheritance)	Somatic Reversion*Cell Type & Detection*	Immunological Findings*T/B/NK counts; Ig levels*	Clinical Features*Infections; autoimmunity; malignancy; therapy response*	Follow-up/Outcome	Reference
**Male infant (onset ~3mo – dx 2 yr)**	**c.343T>C, p.Cys115Arg** (X-linked; mother carrier)	**Yes:** Revertant *T cells* (mutation present in granulocytes/B-cells but *not T-cells*), indicating a reversion in a T-cell progenitor. Detected by genomic sequencing of sorted cells.	**T:** Present (autologous; oligoclonal); **B:** Normal count (dysfunctional); **NK:** Absent (T^+^B^+^NK^−^ phenotype). **Ig:** Low IgG/A; poor specific antibody responses.	**Severe recurrent infections** in infancy (failure to thrive, pneumonias). *No maternal T-cell engraftment.* Survived beyond infancy due to some T-cell function. No malignancy; no autoimmunity reported.	Managed with antibiotics/IVIG; no HSCT by report. Alive at 2 years old with partial immunity.	NEJM 1996 (Stephan et al.) (DOI: 10.1056/NEJM199611213352104)
**Male infant (onset ~2mo – dx 1 yr)**	**c.664C>T, p.Arg222Cys** (X-linked; maternal carrier)	**Yes:** Multiple independent reversion events in *T cells*. Both CD4^+^ and CD8^+^ T-cell populations contain revertant clones. Detected by sequencing (mixed wild-type/mutant IL2RG in T cells).	**T:** Low-to-normal count (with **CD4:CD8 ratio** skewed by expanded revertant T clones); **B:** Normal count (poor function); **NK:** Absent (T^+^B^+^NK^−^). **Ig:** Low IgG; poor vaccine responses.	**Opportunistic infections** in early infancy (thrush, PCP pneumonia). Improved once autologous T cells expanded; later recurrent sinopulmonary infections. No autoimmunity or malignancy reported.	Received IVIG and antimicrobial prophylaxis. Delayed definitive therapy; alive at 2 years old with partial T-cell function.	J Clin Immunol 2012 (Kawai et al.) (DOI: 10.1007/s10875-012-9684-1)
**Male child (onset infancy – dx 6 yr)**	**c.655A>T, p.Tyr219Asn** (X-linked; familial in cousins)	**Yes:** Somatic back-mutation in a *common T progenitor*. ~50% of T cells (both CD4^+^ & CD8^+^) carry wild-type IL2RG sequence. Detected by Sanger sequencing of sorted T-cell subsets. No reversion in B or myeloid cells (mutation found in granulocytes/B-cells).	**T:** Low-normal count (progressive **CD8^+^ expansion** → inverted CD4:CD8 ratio); **B:** Normal count (low class-switched memory B cells); **NK:** Present (near-normal count, partially functional). **Ig:** Normal total IgG but **absent specific antibodies** (no vaccine responses).	**Recurrent bacterial infections** from infancy (otitis, pneumonia); chronic lung damage (**bronchiectasis**) by childhood. Extensive **molluscum contagiosum** skin viral infections. No malignancy; no overt autoimmunity. Infections improved on IVIG and prophylaxis.	Treated with monthly IVIG and antibiotics from age 6. **Clinical improvement** over 7-year follow-up (only one pneumonia; wart lesions eventually regressed). Alive at 13 years with stable partial immunity (no HSCT performed).	Haematologica 2013 (Kuijpers et al.) (DOI: 10.3324/haematol.2012.077511)
**Male child (onset 1 yr – dx 9 yr)**	**c.172C>A, p.Pro58Thr** (de novo)	**Yes:** Somatic mosaicism in *CD8^+^ T cells and NK cells*. Sanger sequencing showed wild-type allele in CD8 T cells and CD56^+^ NK cells (but not in CD4 or B cells). Implies independent reversion in a late lymphoid progenitor. Detected by whole-exome sequencing and targeted deep sequencing.	**T:** Moderate T lymphopenia with **preferential CD8^+^ T-cell recovery** (revertant CD8 clones); low naïve CD4^+^ T cells. **B:** Present (normal count, mutated allele; poor class-switching). **NK:** Present (partial function due to revertant subset). **Ig:** Low IgG (hypogammaglobulinemia). Mitogen T-cell proliferation was decreased.	**Recurrent infections** starting at 1 year (e.g. pneumonias, thrush). **Chronic respiratory infections** led to bronchiectasis. No live-vaccine complications noted. No malignancy; no autoimmunity reported.	**Atypical X-SCID phenotype:** survived to school-age without transplant. After genetic diagnosis, underwent curative HSCT (outcome not detailed). Identified as the *7th reported case* of IL2RG reversion SCID.	J Clin Immunol 2015 (Okuno et al.) (DOI: 10.1007/s10875-015-0202-0)
**Male adolescent (onset childhood – dx 23 yr)**	**c.260T>C, p.Leu87Pro** (X-linked; inherited – mother carrier)	**Yes:** Somatic reversion in a *common lymphoid progenitor*. **All autologous T cells** express wild-type IL2RG (functional γc), and a subset of bone-marrow derived B cells are revertant. Detected by sequencing (mixed mutant/wild-type reads in PBMC, with γc^+^ T cells). NK cells remained mutant (NK-deficient).	**T:** Near-normal count of polyclonal T cells (CD3^+^ ~1200/µL) with low CD4^+^ (persistently CD4 lymphopenic); **B:** Low B-cell count and *no isotype switching* (IgM^+^ only); **NK:** Absent (T^+^B^+^NK^−^ phenotype). **Ig:** IgG <200 mg/dL (marked hypogammaglobulinemia). No vaccine antibody responses.	**CVID-like presentation:** recurrent sinopulmonary infections from age 5 years, requiring IVIG. **Granulomatous skin lesions** developed in adulthood (non-caseating granulomas with T-cell infiltrates). No lymphoma; no autoimmune disease. Partial immune function (revertant T cells) prevented opportunistic infections.	Managed as CVID for two decades (IVIG + antibiotics). Diagnosis revised at 23 years when genetic testing found IL2RG mutation. No curative transplant given the relatively mild course. At last follow-up (age 27) patient clinically stable on IG replacement.	J Clin Immunol 2015 (Hsu et al.) (DOI: 10.1007/s10875-015-0174-0)
**Male child (onset ~2 yr – dx 11 yr)**	**c.172C>T, p.Pro58Ser** (de novo)	**No:** *Hypomorphic* mutation with no detected reversion (mutant IL2RG in all cell lineages). γc protein expressed on lymphocytes but mislocalized (ER/Golgi accumulation). Functional assays confirmed impaired IL-2/IL-21 signaling despite normal IL2RG sequence in all cells.	**T:** Normal total lymphocyte count (age-matched), but **skewed subsets** – low naïve T cells, reduced pDCs. **B:** Normal count (reduced memory B pool); **NK:** Normal count. **Ig:** Near-normal Ig levels but **poor antibody responses** (no vaccine immunity). T-cell proliferation to IL-2 was reduced.	**Atypical X-CID: Recurrent upper & lower respiratory infections** from age 2, progressing to **bronchiectasis** by adolescence. Also suffered **reactive arthritis** (autoimmune arthropathy triggered by infections). No chronic viral warts or malignancies.	Started IVIG and prophylactic antibiotics; pulmonary clearance therapies for bronchiectasis. Not transplanted as of report (partial immune function). Ongoing follow-up into adolescence with multidisciplinary care.	J Clin Immunol 2020 (Tuovinen et al.) (DOI: 10.1007/s10875-020-00745-2)
**3 brothers (onset childhood – dx ages 19, 15, 9)**	**c.458T>C, p.Ile153Thr** (X-linked; mother carrier)	**Yes:** *Somatic reversion in T lymphocytes.* All three had a fraction of T cells with normal IL2RG sequence. **CD3^+^ CD4^+^ and CD8^+^ T cells** showed restored surface CD132 (γc) expression with partial function. Detected by Sanger sequencing of sorted subsets and pSTAT5 assays (mosaic pSTAT5^+ T-cell fraction). No reversion in B or NK cells (mutant IL2RG in those).	**T:** Low–normal total T counts (*CD4^+^ lymphopenia*, **CD8^+^ expansion**) – inverted CD4:CD8 ratios. Preserved naive TREC^+ output in revertant cells (TREC low-normal). **B:** Normal counts (impaired class-switch – low IgG/A MBC). **NK:** Normal count (reduced NK cytotoxic function). **Ig:Dysgammaglobulinemia** – low IgG with high IgM; poor specific antibody formation.	**CVID phenotype:** all three presented with **recurrent pneumonia/bronchitis** in childhood and chronic **sinopulmonary infections**, leading to bronchiectasis on CT in the eldest. Extensive **cutaneous warts** (HPV) on hands and feet in all brothers due to T/NK dysfunction. No autoimmune disease or lymphoproliferative malignancy observed.	Long-term IgG replacement and antimicrobial prophylaxis in all three, with clinical improvement. None has undergone HSCT to date (parents declined immediate transplant given partial immune correction). Eldest (age 19) is in pulmonary rehab for bronchiectasis; younger siblings are clinically stable with preventative care.	Front Immunol 2022 (Hou et al.) (DOI: 10.3390/genes13010035)
**2 brothers (onset infancy – dx neonate & 4 yr)**	**Large IL2RG genomic duplication** (exon duplication) (X-linked; mother carrier)	**Yes:** *Spontaneous genetic rescue by deletion of the duplicated segment.* **Sibling A:** Mosaic reversion in *T and NK cells* (subpopulation with normal IL2RG, others with duplication). **Sibling B:** Similar somatic correction noted. Detected by comparative genomic analysis and drop-digital PCR on sorted cells (demonstrating mosaic loss of the duplication in lymphocytes).	**T:** Sibling A – **T^+^ (low)**: had autologous T cells (mixed revertant and mutant), allowing some thymic function; Sibling B – **T^−^** (initially) on newborn screen, then **T^+^** after revertant clone expansion. **B:** B cells present (duplication allele); **NK:** Sibling A – low, partly revertant; Sibling B – absent initially. **Ig:** Agammaglobulinemia in infancy; low IgG later (on IVIG).	**Sibling A (index, 4 yrs):** Failure to thrive and recurrent **viral pneumonias** in infancy; diagnosed at age 4 with *combined immunodeficiency*. **Sibling B (newborn):** Identified via abnormal SCID screening (low T cells); initially severe infections were prevented by protective isolation. Both showed improvement in T-cell counts over time (suggesting proliferative advantage of revertant cells). No malignancy; no autoimmunity in either.	**Sibling A:** Underwent **hematopoietic stem cell transplant** at 5 years (due to chronic lung disease pre-diagnosis); doing well post-HSCT. **Sibling B:** Given partial T-cell reconstitution from reversion, a decision on HSCT was delayed – he remains on IVIG and prophylaxis, closely monitored at age 1. Both siblings are alive and under follow-up.	J Clin Immunol 2023 (dela Cruz et al.) (DOI: 10.1007/s10875-023-01557-w)
**Male adult (onset 4 yr – dx 34 yr)**	**c.172C>A, p.Pro58Thr** (de novo)	**No:** *Hypomorphic* IL2RG variant, **no reversion** (mutant allele present in T, B, NK). Normal γc surface expression but **reduced JAK-STAT signaling**. Functional tests showed low pSTAT5 in CD4^+^ T cells (and B cells) after IL-2/IL-21 stimulation. NK degranulation also impaired.	**T:** Persistent **CD4 lymphopenia** (CD4 ~300/µL) since childhood; total T and NK counts low-normal. **B:** Normal count (slightly low memory B subset). **NK:** Present (functional defect). **Ig:** Chronically low IgG and IgA (requiring IgRT); poor antibody responses (lost post-vaccine titers). T-cell mitogen responses remained surprisingly intact (near-normal proliferation to PHA).	**“CVID-like” immunodeficiency:** recurrent **sinopulmonary infections** from age 4 (requires continuous IVIG). Chronic **giardiasis** and enteroviral meningoencephalitis in teens (due to low IgG). No granulomas or lymphomas. Notable **CD4 T-cell decline** over decades but patient maintained in relatively stable condition.	Managed as CVID for >30 years. **Genetic diagnosis only made at age 34** via NGS panel. After diagnosis, patient counseled on curative options; currently continuing IVIG and antimicrobials. Plans for gene therapy trial enrollment given residual T-cell immunity and HSCT risks at adult age.	Front Immunol 2025 (González-Torbay et al.) (DOI: 10.3389/fimmu.2025.1544863)

Bold text indicates key distinguishing clinical, immunological, or outcome features highlighted for emphasis.

Table 1. Published cases of IL2RG deficiency presenting with CVID-like features or somatic reversion. The table includes age of symptom onset and diagnosis, IL2RG mutations, immune phenotype, type of somatic reversion (if applicable), clinical manifestations, and follow-up where available.

### Genotype–phenotype correlations of *IL2RG* mutations

**Figure 2:**
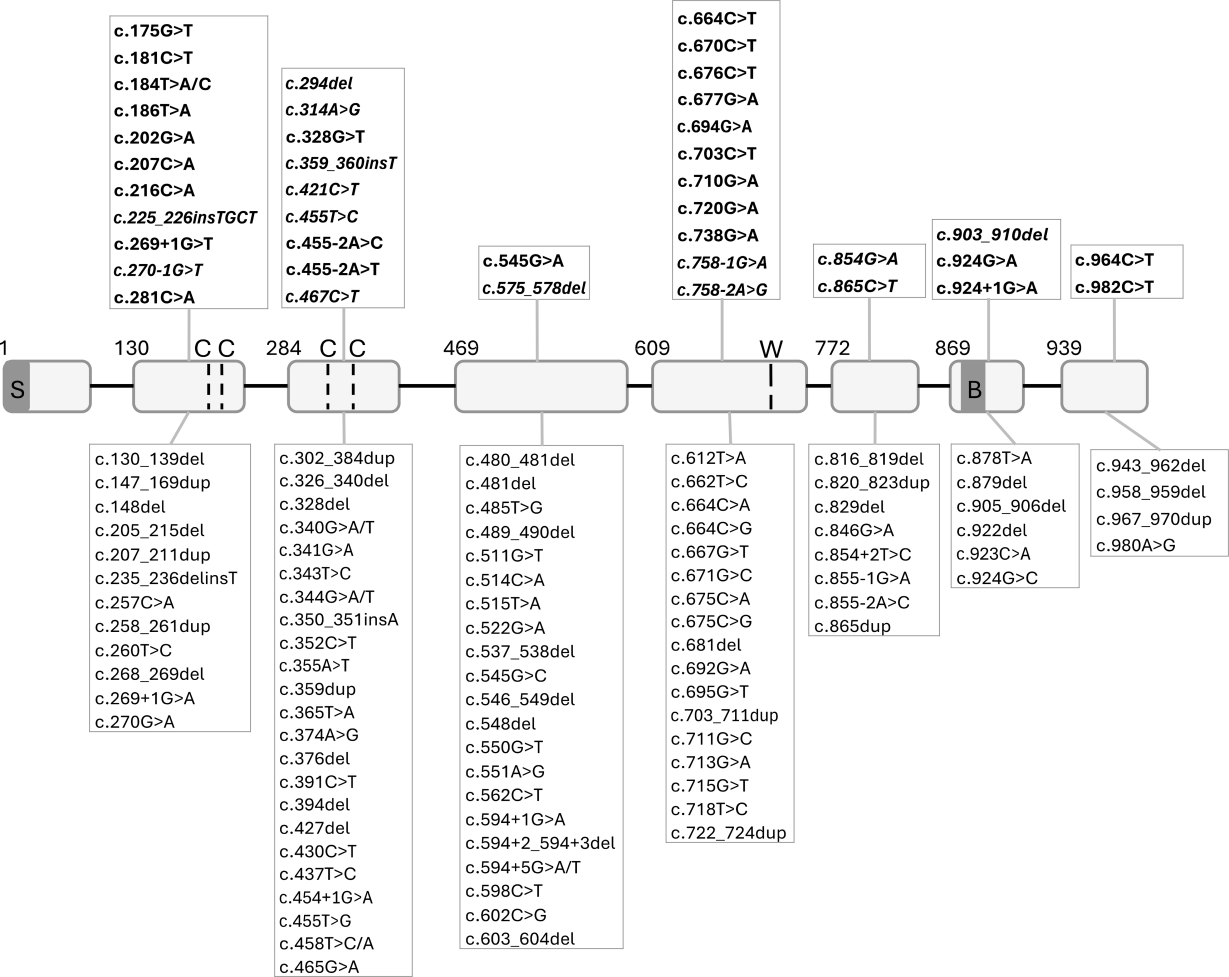
IL2RG gene exons (NM_000206.3) showing pathogenic/likely pathogenic variants. The upper panels present the curated pathogenic variants by the ClinGen SCID VCEP (Regular) and the pathogenic/likely pathogenic variants with more than 2 submissions in ClinVar database (Italics). The bottom panels present the pathogenic/likely pathogenic variants with a single submission in ClinVar database. (S: Signaling sequence; C: Conserved cysteine residues, W: WSXWS box; B: box1/box2 domain).

Hundreds of *IL2RG* gene variants have been catalogued, and genotype–phenotype studies are beginning to elucidate which mutations permit residual function (**Figures 1**, **2**, **Tables 2**, **3**, **Supplementary Table 1**). Null mutations invariably cause classic SCID with no γ_c_ protein and a T−B+NK− phenotype (1, 4). For instance, a complete *IL2RG* deletion or a premature stop codon in the extracellular domain leads to absence of surface CD132 and a classic SCID presentation in infancy (4). Missense mutations, depending on their location and effect, range from null-like to hypomorphic. Missense changes disrupting the highly conserved cysteine residues or WSXWS motif in the extracellular domain usually abrogate proper folding and surface expression of γ_c_, behaving like null mutations (4). In contrast, missense mutations in less critical regions or those that only partially impair cytokine signaling can produce leaky phenotypes. Cytoplasmic domain mutations are a notable category: changes in exon 7–8 that truncate or alter the intracellular tail may allow the receptor to be expressed on the cell surface but impair its signaling capacity (particularly JAK3 binding). For example, the nonsense mutation p.Arg328* in exon 8 (truncating 42 amino acids of the cytoplasmic tail) was reported in a 16-year-old boy with atypical X-SCID (4). That mutant γ_c_ could still form receptor complexes, but had a reduced JAK3 binding site, leading to partial signaling; immunologically the patient had very low T cells (T^low^ B^+^ NK^+^ phenotype) rather than T-null, correlating with residual IL-2/IL-7 responses on testing (4). Generally, truncations after the transmembrane region tend to be hypomorphic (since some receptor reaches the surface), whereas truncations or missense in the extracellular binding domains tend to be more severe.

**Table 2:** Pathogenic/Likely pathogenic variants in the IL2RG gene curated by the the ClinGen SCID VCEP.

Variant	Protein	dbSNP ID	Germline classification	ACMG/AMP criteria	Comment
c.175G>T	(p.Glu59*)	rs2092262517	Likely pathogenic	PVS1 PM2_Supporting	It is predicted to cause a premature stop codon in biologically-relevant-exon 2/8 leading to nonsense-mediated decay. It has not been reported in the literature in individuals with SCID yet.
c.181C>T	(p.Gln61*)	rs1569480082	Likely pathogenic	PVS1 PM2_Supporting	It is predicted to cause a premature stop codon in biologically-relevant-exon 2/8 leading to nonsense-mediated decay. This variant is reported in a patient with SCID (PMID: 35874699).
c.184T>A	(p.Cys62Ser)	rs1602289649	Likely pathogenic	PP4_Moderate PM1_Strong PM2_Supporting	This variant affects a conserved cysteine residue Cys62. The variant was found in a male patient with SCID (PMID : 35874699).
c.184T>C	(p.Cys62Arg)	rs1602289649	Pathogenic	PS4_Supporting PM5 PM1_Strong PP4_Moderate PM2_Supporting	This variant affects a conserved cysteine residue Cys62. Other variants that disrupt this residue have been observed in individuals with IL2RG-related conditions (PMID: 8605324, 25843602), which suggests that this may be a clinically significant amino acid residue. It is predicted that this variant may create or strengthen a splice site for mRNA splicing. This missense change has been observed in individual with clinical features of severe combined immunodeficiency (PMID: 27484032).
c.186T>A	(p.Cys62*)	rs111033619	Pathogenic	PP4 PVS1 PM2_Supporting	It is predicted to cause a premature stop codon in biologically-relevant-exon 2/8 leading to nonsense-mediated decay. The variant was found in a male patient with SCID (PMID: 8462096).
c.202G>A	(p.Glu68Lys)	rs1057520644	Pathogenic	PP4_Moderate PS4 PM2_Supporting	This variant is found in multiple unrelated affected probands (PMIDs: 9058718, 24534054,11129345, 32477911,31456805, 27484032,31031743,30778343).
c.207C>A	(p.Tyr69*)	rs1282558220	Pathogenic	PVS1 PM2_Supporting PP4	It is predicted to cause a premature stop codon in biologically-relevant-exon 2/8 leading to nonsense-mediated decay. The variant was found in a male patient with SCID (PMID: 19398866).
c.216C>A	(p.Cys72*)	rs2147750927	Likely pathogenic	PVS1 PM2_Supporting	It is predicted to cause a premature stop codon in biologically-relevant-exon 2/8 leading to nonsense-mediated decay. It has not been reported in the literature in individuals with SCID yet.
c.269+1G>T		rs2092262300	Pathogenic	PVS1 PM2_Supporting PP4_Moderate	It is expected to cause skipping of a biologically relevant exon 2 resulting in a frameshift (p.Asp39Glyfs*57) leading to nonsense mediated decay. The variant was found in a male patient with SCID (PMID: 33628209).
c.281C>A	(p.Ser94*)	rs775704953	Pathogenic	PM2_Supporting PVS1 PP4	It is predicted to cause a premature stop codon in biologically relevant exon 3/8, leading to nonsense-mediated decay. The variant was found in a male patient with SCID (PMID: 23250629).
c.328G>T	(p.Glu110*)	rs2147750382	Pathogenic	PVS1 PM2_Supporting PP4_Moderate	It is predicted to cause a premature stop codon in biologically relevant exon 3/8, leading to nonsense-mediated decay. The variant was found in a male patient with SCID (PMID: 10794430).
c.455-2A>C		rs2092260728	Pathogenic	PVS1 PM2_Supporting PS1	It is predicted to cause skipping of biologically relevant exon 4 (SpliceAI score 0.59), resulting in a frameshift leading to nonsense-mediated decay. It has not been reported in the li*ature in individuals with SCID yet, however, it is located in the splice consensus sequence at the same nucleotide position as another pathogenic variant, c.455-2A>C (PMID 28747913).
c.455-2A>T			Pathogenic	PVS1 PP4 PM2_Supporting	It is predicted to cause skipping of biologically relevant exon 4 (SpliceAI score 0.59), resulting in a frameshift leading to nonsense-mediated decay. The variant has been identified in one male individual who was diagnosed with SCID (PMID 28747913).
c.545G>A	(p.Cys182Tyr)	rs1064794027	Pathogenic	PS4 PM2_Supporting PP4_Moderate	Another missense variant Cys182Arg in the same codon has been reported (PMID: 10794430). This variant has been reported in at least 6 probands (PS4_met, 5pts)(PMIDs: 25109802,22039266,18688286,35482138, 21865537,18728247).
c.664C>T	(p.Arg222Cys)	rs111033618	Pathogenic	PP4_Moderate PS4 PM2_Supporting PP1_Strong	It has been reported in several (atypical) SCID cases (PMID: 29948574). At least 15 additional male X-SCID patients have been reported (PMIDs: 25042067, 16227049, 10794431, 7557965) with this hemizygous variant.
c.670C>T	(p.Arg224Trp)	rs869320658	Pathogenic	PS4 PP4 PS3_Supporting PM1_Strong PM2_Supporting	The variant affects CpG dinucleotides at c.670C, which is defined as a mutational hotspot (PMID 7668284). Surface expression of the IL-2 receptor gamma chain in patient B cells showed that the variant causes decreased surface localization of the protein, indicating that this variant impacts protein function (PMID 9058718). It has been observed in at least 10 male probands with SCID (PMIDs 28747913, 21184155, 10792291, 9633906, 9058718, 9049783).
c.676C>T	(p.Arg226Cys)	rs869320659	Pathogenic	PS3_Supporting PM1_Strong PP4 PS4 PM2_Supporting PM6	It resides within a CpG dinucleotide region, cDNA 676-677 encoding amino acid R226, of IL2RG that is defined as a mutational hotspot (PMID: 7668284). At least one male proband in the literature presents the diagnostic cri*ia for SCID/Leaky SCID/Omenn syndrome (PMID: 14966353, 9058718, 11129345, 22936741, 7668284). Published functional studies demonstrate a damaging effect on protein function (PMID: 7632950).
c.677G>A	(p.Arg226His)	rs869320660	Pathogenic	PM2_Supporting PS3_Supporting PS4 PM1_Strong PM5 PP4_Moderate	This variant affects CpG dinucleotides at c.677G, which is defined as a mutational hotspot (PMID 7668284). Experimental studies have shown that this missense change affects IL2RG function (PMID: 9058718, 11213805, 16760466). The variant has been observed in at least 9 probands with SCID/Ommen Syndrome (PMIDs 7668284, 9058718, 17598841, 21184155).
c.694G>A	(p.Gly232Arg)	rs1569479909	VUS/Likely Pathogenic	PP4 PM2_Supporting PM5	This variant has been identified in a male individual with T cell lymphopenia who met WHO critical for SCID (PMID: 9058718, 8961626). This variant has also been reported in a second male individual with a reported phenotype of SCID (PMID: 28747913). A different nucleotide change (c.694G>C) that results in the same missense change as (p.Gly232Arg) was reported in a patient with a suspected inborn error of immunity (PMID: 35874699). Two additional variants c.695G>A and c.695G>T resulting in different missense changes at this same codon (p.Gly232Glu and p.Gly232Val, respectively) were reported in individuals with suspected primary immunodeficiencies (PMID: 28359783, 33942430).
c.703C>T	(p.Gln235*)	rs1556330249	Pathogenic	PM2_Supporting PVS1 PP4	It is predicted to cause a premature stop codon in biologically relevant exon 5/8, leading to nonsense-mediated decay. At least one male proband in the li*ature presents the diagnostic critical for SCID/Leaky SCID/Omenn syndrome (PMID: 11129345).
c.710G>A	(p.Trp237*)	rs193922350	Pathogenic	PVS1 PM2_Supporting PP4	It is predicted to cause a premature stop codon in biologically relevant exon 5/8, leading to nonsense-mediated decay. At least one male proband in the li*ature presents the diagnostic critical for SCID/Leaky SCID/Omenn syndrome (PMID: 28702026, 21184155).
c.720G>A	(p.Trp240*)	rs1556330234	Pathogenic	PP4_Moderate PVS1 PM2_Supporting	It is predicted to cause a premature stop codon in biologically relevant exon 5/8, leading to nonsense-mediated decay. A different variant, c.719G>A, which results in the same truncating effect (p.Trp240*) on the protein and absent IL2RG expression, has been observed in a patient affected with classic X-linked SCID. In addition, fourteen unrelated patients with classic X-linked SCID have been observed with truncating mutations in exon 5 showing nonsense-mediated decay and absence of IL2RG mRNA (PMID: 9058718).
c.738G>A	(p.Trp246*)	rs2147748109	Pathogenic	PP4 PM2_Supporting PVS1	It is predicted to cause a premature stop codon in biologically relevant exon 5/8, leading to nonsense-mediated decay. At least one male proband in the literature presents the diagnostic cri*ia for SCID/Leaky SCID/Omenn syndrome (PMID: 10794430, 28109013).
c.924+1G>A		rs886041333	Pathogenic	PVS1 PM2_Supporting PP4_Moderate	It disrupts the canonical splice donor site in intron 7, which is predicted to cause skipping of exon 7 and a frameshift in exon 8 (the final exon) with a stop loss and elongation of the protein by 31 amino acids (p.Thr286Profs*57). While the critical cytoplasmic domain is not truncated it is entirely al*ed and considered to be functionally lost, therefore loss of the critical cytoplasmic domain. Additionally, the splice effect was evaluated in patient-derived cells, where trace amounts of an abnormally large IL2RG mRNA were detected, containing intronic sequences due to aberrant splicing (PMID: 9058718). At least one male patient in the li*ature presents a specific T-B+NK- lymphocyte subset profile (PMID: 35812426).
c.924G>A	(p.Ser308=)	rs2092255386	Likely pathogenic	PP1 PP3 PM4 PM2_Supporting PP4_Moderate	This synonymous variant occurs at the final nucleotide of exon 7 and is predicted to impact the splice consensus sequence (SpliceAI score 0.95) with loss of the donor splice site. It has been reported to segregate with X-SCID through 3 affected segregations. Validation of this splicing defect in IL2RG mRNA analysis was performed. Skipping of exon 7 was detected which would cause a frameshift in exon 8 with a stop loss and elongation of the protein by 31 amino acids (PMID: 30850927, PMID: 9058718).
c.964C>T	(p.Gln322*)		Pathogenic	PVS1 PP4 PM2_Supporting	It is not predicted to cause nonsense-mediated decay. At least one male patient in the literature presents the diagnostic critical for SCID/Leaky SCID/Omenn syndrome (PMIDs: 33628209).
c.982C>T	(p.Arg328*)	rs1064793347	Pathogenic	PVS1 PM2_Supporting PP4_Moderate	It is not predicted to cause nonsense-mediated decay, as it is located in a critical region for protein function. At least one male proband in the li*ature presents the diagnostic critical for SCID/Leaky SCID/Omenn syndrome. Published functional studies demonstrate a damaging effect on protein function, with impaired binding of JAK3 to the common gamma chain (PMID: 31799703).

**Table 3:** Pathogenic/Likely pathogenic variants in the IL2RG gene with more than 2 submissions in ClinVar database.

Variant	Protein	dbSNP ID	Germline classification	ACMG/AMP criteria	Comment
c.225_226insTGCT	(p.Ser76delinsCys*)	rs1602289631	Likely pathogenic	PVS1 PM2_Supporting	This variant is predicted to cause loss of normal protein function either through protein truncation or nonsense-mediated mRNA decay.
c.270-1G>T		rs193922346	Pathogenic	PVS1 PS4_moderate PS3_Supporting PM2_Supporting	It is predicted to cause abnormal gene splicing. Experimental studies have shown that disruption of this splice site results in altered mRNA splicing and is expected to lead to the loss of protein expression. This variant has been observed in affected male with SCID (PMID: 28359783).
c.294del	(p.Val99fs)	rs2092261618	Pathogenic	PVS1 PM2_Supporting PS4	It is expected to result in an absent or disrupted protein product. This variant has been observed in many individuals affected with SCID (PMID: 10794430).
c.314A>G	(p.Tyr105Cys)	rs193922347	Pathogenic	PS4 PM2_Supporting PP4_Moderate	This variant has been observed in individuals with severe combined immunodeficiency (PMID: 8961626, 10444186, 33628209)
c.359_360insT	(p.Lys120fs)	rs2092261347	Pathogenic	PVS1 PM2_Supporting PS4	It is predicted to cause a truncation of the encoded protein or absence of the protein due to nonsense mediated decay. It has been reported in the literature in individuals affected with SCID (PMID: 10794430).
c.421C>T	(p.Gln141*)	rs1556330713	Likely pathogenic	PVS1 PM2_Supporting	It is predicted to cause a premature stop codon in biologically-relevant-exon 2/8 leading to nonsense-mediated decay. It has not been reported in the literature in individuals with SCID yet.
c.455T>C	(p.Val152Ala)	rs193922348	Likely pathogenic	PVS1 PM2_Supporting	This variant has been observed in individual with severe combined immunodeficiency (PMID: 25326637).
c.467C>T	(p.Ala156Val)	rs1057521062	Pathogenic	PS4 PM2_Supporting PP4_Moderate	This variant has been reported to affect IL2RG protein function (PMID: 9885222, 8027558). This variant has been observed in individual(s) affected with X-linked severe combined immunodeficiency (PMID: 9885222, 8027558)
c.575_578del	(p.Asp192fs)		Likely pathogenic	PVS1 PM2_Supporting	It is expected to result in an absent or disrupted protein product.
c.758-1G>A		rs886042051	Pathogenic	PVS1 PM2_Supporting PS4	This sequence change affects an acceptor splice site in intron 5 of the IL2RG gene. It is expected to disrupt RNA splicing and likely results in an absent or disrupted protein product. Disruption of this splice site has been it has been observed in several individuals affected with severe combined immunodeficiency (PMID: 10794430).
c.758-2A>G		rs2147747509	Pathogenic	PVS1 PM2_Supporting PS4	It is predicted to affect mRNA splicing resulting in a significantly altered protein due to either exon skipping, shortening, or inclusion of intronic material. It has been reported in the literature in at least one individual affected with X-Linked Severe Combined Immunodeficiency (PMID: 33412294).
c.854G>A	(p.Arg285Gln)	rs111033617	Pathogenic	PVS1 PM2_Supporting PS4	This variant also falls at the last nucleotide of exon 6, which is part of the consensus splice site for this exon. This variant has been observed in individual(s) with X-linked severe combined immunodeficiency (PMID: 7557965, 9058718, 21184155, 22039266). Variants that disrupt the consensus splice site are a relatively common cause of aberrant splicing (PMID: 17576681, 9536098).
c.865C>T	(p.Arg289*)	rs137852508	Pathogenic	PVS1 PM2_Supporting PS4	It is predicted to cause a truncation of the encoded protein or absence of the protein due to nonsense mediated decay. This variant has been observed in individual(s) with X-linked recessive severe combined immunodeficiency (PMID: 8462096, 8781427).
c.903_910del	(p.Glu302fs)	rs1556329779	Pathogenic	PS4 PM2_Supporting PP4_Moderate	This creates a premature translational stop signal in the last exon of the IL2RG mRNA (p.Glu302Argfs*11). While this is not anticipated to result in nonsense mediated decay, it is expected to disrupt the last 66 amino acids of the IL2RG protein. it is expected to disrupt the C-terminal region of the interleukin receptor common gamma chain, which is known to be critical for proper association with Jak3 (PMID: 7973658, 7973659). Deletions affecting this intracellular region of the protein have been shown to lead to defects in signal transduction, including loss of ability to induce c-myc, c-fos, and c-jun expression (PMID: 7683423).

#### Correlation trends

Recently the ClinGen SCID Variant Curation Expert Group (VCEP) curated the most relevant and well-established variants associated with SCID in *IL2RG* gene (**Table 2**) and refined the 2015 American College of Medical Genetics and Genomics/Association for Molecular Pathology (ACMG/AMP) guidelines for variant classification, adapting them in the context of SCID. By doing so, the ClinGen SCID VCEP was also able to refine the pathogenicity of several *IL2RG* variants (31). Additionally, the ClinVar database included more that 140 pathogenic or likely pathogenic variants related with IL2RG-SCID (**Table 3**, **Supplementary Table 1**). Comparing *unique* IL2RG mutations found that about half of all known pathogenic variants are located in exon 5 (which encodes much of the extracellular domain, including the WSXWS motif) and exon 3 (4). These hotspots tend to contain null variants. In contrast, most hypomorphic mutations reported occur in exons encoding the cytoplasmic tail or membrane-proximal regions (4). For example, exon 7–8 have been associated with atypical SCID (4). Additionally, specific “leaky” missense mutations have been identified: p.Arg222Cys (in the extracellular domain) is a prominent example with variable expressivity (3). Others include p.(Leu162Arg) and p.(Leu306Gln), which have been reported in patients with partial T-cell immunity (3). A novel missense p.Pro58Ser was recently shown to impair IL-2 receptor complex formation and signaling despite normal γ_c_ surface expression, causing an “X-CID” phenotype with bronchiectasis in childhood (3). **Tables 2**, **3** summarize representative IL2RG mutations and their clinical phenotypes.

**Table 2**. Examples of *IL2RG* mutations and associated clinical phenotypes. (References: representative cases from (1, 4, 26, 27, 32, 33).

This genotype–phenotype correlation is not absolute, but certain trends emerge mutations that abolish protein expression or critical structure cause typical SCID, whereas those that leave some residual expression or function (altering signaling efficiency) often result in “combined immunodeficiencies” (CID) of intermediate severity. Recognition of these correlations helps in prognostication and management. For instance, identifying a hypomorphic *IL2RG* mutation in a child with infections might explain a milder course and influence the urgency/timing of HSCT. Conversely, finding a classic null mutation in a seemingly well infant (perhaps detected by screening before illness) should prompt rapid definitive therapy before infections strike.

## Functional evaluation of novel *IL2RG* variants: diagnostic utility and clinical integration

As next-generation sequencing identifies an expanding list of *IL2RG* candidate variants, functional assays have become indispensable for determining pathogenicity. Variants of uncertain significance (VUS), particularly in atypical or late-onset cases, necessitate biological validation to guide management. The most direct and informative approach is phospho-flow cytometry assessing STAT5 phosphorylation following IL-2 or IL-7 stimulation of patient-derived lymphocytes (34). Absence or reduction of pSTAT5 signal supports impaired γ_c_ signaling, a hallmark of pathogenic *IL2RG* mutations. Additional assays cytokine-specific expansion (e.g., IL-15-driven NK expansion), and apoptosis resistance testing. In ambiguous cases or when patient material is unavailable, transfection of wild-type and mutant *IL2RG* constructs into γ_c_-deficient cell lines (e.g., ED-7R or *IL2RG*^–/–^ Jurkat) can reveal differences in surface expression, STAT activation, or JAK3 binding (3).

Importantly, some hypomorphic mutations do not impair surface expression but abrogate downstream signaling—particularly those in the cytoplasmic tail, as seen in p.Arg328* or other exon 7–8 truncations. In such cases, western blotting for phosphorylated STAT5 or JAK3 co-immunoprecipitation may be required. Interpretation should integrate in silico predictions (e.g., PolyPhen, CADD), conservation analysis, and family segregation data (4).

We propose a standardized functional workup of novel IL2RG variants involving (**Box 2**):

- IL-2/IL-7 pSTAT5 assays in patient T cells (or PBMCs)
- CD132 surface expression analysis by flow cytometry
- Proliferation to cytokines (IL-2, IL-7, IL-15)
- Transfection-based modeling in γ_c_-null lines, if needed
- Testing in multiple cytokine contexts (e.g., plasma blast differentiation in response to IL-21 for B-cell signaling relevance)

Given the variable phenotypes—including CVID-like, HLH-triggered, or hyper-IgE presentations—early application of functional testing facilitates correct diagnosis and timely definitive therapy (HSCT or gene therapy), even in adolescents or adults (26).

Key clinical and laboratory red flags for IL2RG-related disease are summarized in **Box 1**.

Box 1 **Diagnostic red flags for IL2RG-related immunodeficiency (X-linked SCID and atypical forms)**

The following clinical and laboratory features should raise suspicion for IL2RG-related immunodeficiency and prompt targeted genetic and functional evaluation:

• **Male infant with severe, recurrent or opportunistic infections** (e.g. *Pneumocystis jirovecii* pneumonia, persistent viral or fungal infections), failure to thrive and lymphopenia in the first months of life.

• **T^-^B^+^NK^-^ immunophenotype** (markedly reduced CD3^+^ T cells, absent or very low NK cells, with normal or increased CD19^+^ B cells), especially in a boy.

• **Very low or undetectable TRECs on newborn screening**, with confirmatory testing showing T-cell lymphopenia and impaired T-cell proliferation to mitogens.

• **BCG-related complications** (disseminated or severe local BCGitis) or prolonged diarrhea after live rotavirus vaccination in early infancy.

• **Evidence of maternal T-cell engraftment**, particularly a memory-skewed (CD45RO^+^, HLA-DR^+^) T-cell phenotype in a neonate who should normally have a naïve T-cell repertoire.

• **EBV-driven hemophagocytic lymphohistiocytosis (HLH) or XLP-like disease** in a boy, especially when associated with T-cell or NK-cell dysfunction and/or a T^-^B^+^NK^-^ or T-low immunophenotype.

• **Male child or young adult labeled as “CVID” or “combined immunodeficiency”** who shows T-cell lymphopenia or dysfunction (low naïve CD4^+^ T cells, poor T-cell proliferation, abnormal γc cytokine responses), dysgammaglobulinemia, and opportunistic or atypical infections.

• **Family history suggestive of X-linked inheritance**, including early male infant deaths, “undefined” severe infections in male relatives, or known SCID in the maternal lineage.

Box 2 *Proposed working features of atypical IL2RG-related X-CID (T-low/T-normal):*

*– Male patient with a pathogenic or likely-pathogenic IL2RG variant predicted or shown to be hypomorphic (partial γc expression and/or signaling).*

*– CD3^+ T-cell counts above the classical SCID range (often >100–200/µL), but with reduced naïve CD4^+CD45RA^+ T cells and/or abnormal TCR repertoire.*

*– Presence of B cells and NK cells (B^+NK^+/low), with impaired functional responses (e.g. reduced cytokine-induced STAT5 phosphorylation, poor vaccine-specific antibodies).*

*– Clinical phenotype of recurrent and/or severe infections and/or immune dysregulation (autoimmunity, lymphoproliferation, granulomatous disease), usually with survival beyond early childhood without immediate HSCT.*

*– Exclusion of alternative causes of combined immunodeficiency.*

## Management and treatment of IL2RG-related immunodeficiencies

### General principles

All *IL2RG*-related immunodeficiencies – from classic X-SCID to atypical forms – are fundamentally disorders of the adaptive immune system that, in most cases, require definitive immune reconstitution. The mainstay of curative treatment is hematopoietic stem cell transplantation (HSCT), which has been used for X-SCID for decades with high success. More recently, gene therapy has emerged as a promising definitive treatment. In addition, supportive measures are critical to protect patients from infection and other complications while definitive therapy is pursued. Because severity can range, the management must be individualized based on the patient’s immune function; however, even “mild” IL2RG deficiency can worsen or have life-threatening events, so aggressive therapy is justified. We discuss supportive care, transplantation, and gene therapy in turn.

### Supportive care and infection prophylaxis

Once *IL2RG* deficiency is suspected or diagnosed, stringent infection prophylaxis and environmental management are instituted immediately (35). Infants should be kept in protective isolation (ideally in a positive-pressure HEPA-filtered room in hospital, or at home with infection control precautions) (35). Strict hand hygiene and avoidance of sick contacts are mandatory (35). Breastfeeding from CMV-seropositive mothers should be avoided (to prevent CMV transmission) or breast milk is pasteurized, and CMV-negative blood products are used for transfusions (36). Trimethoprim-sulfamethoxazole prophylaxis is given by 4–6 weeks of age to prevent *Pneumocystis jirovecii* pneumonia (35, 36). Antifungal prophylaxis (e.g. fluconazole or nystatin) is often started to prevent candida infections (36). Viral prophylaxis (e.g. acyclovir) may be used if there is risk of HSV or if maternal serologies warrant (CMV). Importantly, live vaccines are contraindicated in these patients – this includes rotavirus (typically given at 2 months), which must be withheld if SCID is diagnosed by newborn screening, as well as BCG, oral polio, measles-mumps-rubella, varicella, etc. If the infant already received live vaccines (e.g. in countries with BCG administration at birth), monitoring and empiric treatment (e.g. anti-mycobacterial therapy for BCG) is indicated (37). Intravenous immunoglobulin (IVIG) should be started promptly to provide passive immunity since the patient cannot produce antibodies (36). IVIG can significantly reduce bacterial infection risk and is a bridge to transplant. Nutritional support is also crucial – many SCID infants have failure to thrive from chronic diarrhea or infections, so high-calorie diets and if needed nasogastric feeding are used to optimize their condition pre-transplant. If an atypical older patient has some immune function, prophylaxis regimens are tailored to their specific deficits (e.g. if T cells are present but B cells impaired, IVIG and perhaps only certain prophylaxis might be needed). Nonetheless, any IL2RG-deficient patient should be treated as immunocompromised, with aggressive antibiotics at any sign of infection and close monitoring for opportunistic infections.

### Hematopoietic stem cell transplantation

Allogeneic HSCT has been the standard, life-saving treatment for X-SCID for over 50 years. Transplantation introduces a donor stem cell population that can differentiate into T, B, and NK cells with functional IL2RG, thereby reconstituting the immune system. Outcomes of HSCT: Success rates for HSCT in SCID are now very high, especially when performed early in life and before infections ensue. Interestingly, one recent analysis found that pre-transplant infection status was a stronger predictor of survival than age at transplant (2). Infants transplanted infection-free had ~90% survival even if older than 3.5 months, whereas infected infants had worse outcomes regardless of age. This suggests that with newborn screening (minimizing infections), even slightly older infants do well, and that aggressive infection prevention/treatment is paramount (2). Multicenter studies have reported 5-year survival of 80–95% for SCID infants transplanted by 3–4 months of age and prior to infection onset (38). A recent study of the Primary Immune Deficiency Treatment Consortium (PIDTC) has shown that for children with SCID identified by newborn screening since 2010, 5-year survival rate after HSCT is 92.5%, better that of children identified by positive family history or clinical signs of disease in the same period (39). The European SCETIDE cohort (2006–2014) reported a 2-year overall survival of 87.1% for IL2RG-deficient SCID patients (n=87) after HSCT (2). For IL2RG SCID in particular, outcomes are excellent with an HLA-matched sibling donor (MSD) – survival ~90–95% in most series (2, 38, 39). Even with alternative donors, early transplant yields favorable results, with 5-year survival rates exceeding 80% for patients transplanted since 2010 (39), and superior to what observed after HSCT for ADA deficiency and DNA repair defects (39, 40). Donor source: If an MSD is available, that is ideal. Matched sibling transplants historically were done without any conditioning chemotherapy and still achieved T-cell engraftment because SCID infants lack host T cells to reject the graft (38). T cells from the donor marrow populate the thymus and restore cellular immunity, often sufficient to protect the child. However, in the absence of conditioning, host B-cell precursors survive (they are not eradicated) and thus the B cells remain patient-derived and IL2RG-deficient. Consequently, many patients transplanted without conditioning have permanent B-cell dysfunction requiring lifelong IVIG (41, 42). This trade-off (avoiding chemotherapy toxicity at the cost of B-cell function) was historically acceptable. In recent years, though, practices have shifted: even MSD recipients may receive minimal conditioning to support donor B-cell engraftment and immune self-sufficiency (43). For patients without an MSD, other graft sources include a matched unrelated donor (MUD), haploidentical parental donor (typically T-cell depleted marrow or peripheral stem cells from father or mother), or umbilical cord blood. In the SCETIDE analysis, HLA-matched donors (related or unrelated) had superior survival compared to mismatched family donors: 2-year OS was ~90% with matched donors vs ~70–77% with mismatched unrelated or haploidentical donors (2). Still, even haploidentical transplants can cure SCID; centers often proceed with a haploidentical transplant rather than wait for a MUD, to minimize time-related risk of infections. Conditioning regimens: One of the biggest evolutions in SCID transplant in the last decade is the nuanced use of conditioning. Because SCID infants cannot reject grafts (no T cells), transplants can “take” without conditioning – but as noted, donor B-cell engraftment is poor without at least some marrow conditioning (43). Studies from the Primary Immune Deficiency Treatment Consortium (PIDTC) showed that SCID patients who received even low-dose busulfan conditioning had much higher rates of B-cell reconstitution (and eventually could discontinue IVIG) compared to those who received no conditioning (43). Moreover, even T cell reconstitution was superior in X-SCID patients who received chemotherapy-based conditioning for HSCT from donors other than matched siblings (40). Furthermore, also the risk of graft failure and the need of second transplant are reduced when conditioning is used (40). Thus, the trend is toward using reduced-intensity conditioning (RIC) in HSCT for X-SCID. This has led to a majority of recent IL2RG SCID survivors becoming immunoglobulin-independent with full T and B immunity (40, 43).

### Transplant considerations

For IL2RG SCID, an advantage is that graft-vs-host disease (GVHD) is usually mild. Since patients have no T cells, they cannot reject the graft, and if a T-cell-depleted graft is used, GVHD is minimized. Matched sibling grafts (unmanipulated) can cause some GVHD, but it’s often manageable with immunosuppression. Until recently, haploidentical transplants have been typically T-depleted to avoid GVHD, but in recent years an alternative approach has been frequently used, that is based on the transfusion of bone marrow (or less often, peripheral blood mobilized) stem cells, followed by the administration of cyclophosphamide post-transplant (PT-CY) to achieve depletion of alloreactive T cells (44). Immune reconstitution monitoring is crucial post-transplant: T-cell numbers usually rise by 3–4 months post-HSCT and thymic output (naïve T cells) indicates success. B-cell chimerism should be assessed – patients with donor B cells can often stop IVIG after developing specific antibody responses (43). If B-cell engraftment is absent, long-term IVIG is needed. NK-cell reconstitution is also examined; interestingly, initial engraftment of NK cells is often observed even without conditioning in X-SCID (presumably due to donor NK precursors filling an empty niche), however over time in the absence of conditioning donor-derived NK cells typically disappear from the circulation. Long-term, transplanted X-SCID patients generally have excellent quality of life and immune function, though they require monitoring for late effects. Survivors who received chemotherapy-based conditioning may have risks of endocrinopathies, infertility, or secondary malignancies (43). In a recent study of the PIDTC, chronic and late effects were recorded in 43/153 (28.1%) patients with X-SCID, and were predominantly represented by neurodevelopmental/neurological, dental, pulmonary and hepatic problems (45). A peculiar late complication noted in IL2RG/JAK3 SCID survivors is an increased incidence of wart infections (HPV) on the skin years post-transplant (38). In one cohort, many IL2RG/JAK3 patients developed refractory cutaneous HPV warts ~7 years after HSCT (38). This might relate to incomplete reconstitution of tissue-resident immunity or NK-cell function against HPV. These patients often require aggressive dermatologic care and sometimes donor lymphocyte infusions or antivirals. Late-onset enteric virus infection associated with hepatitis (EVAH) is another complication, that has been mostly observed in patients who did not receive conditioning and failed to develop B-cell immunity (46). In these patients, an expansion of memory CD8+ cells with increased type I and type II interferon signature has been reported. Retransplantation with reduced-intensity conditioning may allow immune reconstitution and viral clearance (46). Nonetheless, the vast majority of transplanted X-SCID patients lead healthy lives, and early HSCT has essentially normalized lifespan.

### Hematopoietic stem cell gene therapy

Because HSCT has been shown to be curative for X-SCID – introducing a correct *IL2RG* gene into the patient’s own hematopoietic stem cells (HSCs) should also reconstitute immunity. A major advantage of gene therapy is that it offers a potential cure without the need for an allogeneic donor and without risking GVHD. The first human gene therapy trial in X-SCID was initiated in Paris in 1999, and soon thereafter also in London, using a γ-retroviral vector to deliver the *IL2RG* cDNA to autologous bone marrow CD34^+^ cells, without using chemotherapy (47). Eighteen of the 20 patients treated with this protocol are still alive, and correction of T cell development with sustained clinical benefit was observed in most patients (48); however, in the absence of conditioning, gene therapy failed to correct B cell function, and two thirds of the patients remain dependent on immunoglobulin replacement therapy (49). Importantly, 6 of the 20 patients developed a form of T-cell acute leukemia 2–14 years after treatment due to vector insertions near oncogenes (LMO2, *CCND2*) *(*49–52) with consequent oncogene transactivation mediated by the enhancer contained in the long terminal repeat (LTR) of the vector. This adverse outcome halted many other gene therapy trials and spurred development of safer vectors. Over the past decade, self-inactivating (SIN) γ-retroviral and lentiviral vectors have been developed to reduce insertional mutagenesis risk (43). Self-inactivating γ-retroviral vectors in which the enhancer contained in the LTR was deleted, and *IL2RG* expression was driven by short version of the elongation factor 1α promoter have proven to be safe (53). As opposed to γ-retroviral vectors, lentiviral vectors offer the advantage of a more random integration of the viral vector DNA into the host’s genome, and have therefore replaced γ-retroviral vectors in clinical trials for SCID. Very promising results have been obtained with lentiviral gene therapy for X-SCID. In a trial conducted at St. Jude Hospital and at the NIH, previously untreated children as well as older children and adults who had failed to achieve immune reconstitution after HSCT for X-SCID, manifested T-cell reconstitution after lentiviral gene therapy, and no leukemia has been observed to date (54). Moreover, use of low dose busulfan prior to gene therapy permitted robust and durable engraftment of gene-corrected HSCs and multilineage reconstitution. In a follow-up study of 8 of the initial 15 older children and infants treated with this vector, discontinuation of IVIG treatment and robust antibody production was documented in 4 patients, indicating successful B-cell reconstitution (54). A similar trial, led by investigators in Boston, have shown excellent T-cell reconstitution with no evidence of clonal expansions (43). For X-SCID patients who lack a suitable transplant donor or who have persistent immune dysfunction after transplant, gene therapy is becoming an increasingly viable option. It is expected that gene therapy for X-SCID will soon move from clinical trial to standard practice, pending long-term safety data and as long as access to gene therapy for rare and ultra-rare diseases is provided (55).

In addition to gene addition, gene editing strategies are being explored. CRISPR/Cas9 technology can be used to precisely correct *IL2RG* mutations in patient HSCs through base editing or insert a normal *IL2RG* cDNA into the endogenous locus or in a safe genomic harbor (like the *AAVS1* locus). Preclinical studies have demonstrated correction of the *IL2RG* defect in X-SCID patient cells using CRISPR-mediated homology-directed repair (56–58). A clinical trial to correct certain IL2RG mutations with base editing is currently open at the NIH (NCT06851767 in https://clinicaltrials.gov). Gene editing holds appeal because it can place the gene under its natural regulatory control, potentially reducing risks of dysregulation inherent to random insertion. Regardless, the future of IL2RG deficiency treatment is likely to feature gene therapy prominently, either via improved vectors or gene editing, reducing the need for allogeneic transplant especially in settings without a matched donor.

### Treatment algorithm

In practice, management of an IL2RG-deficient patient can be summarized in a treatment algorithm:

1. Diagnosis – If newborn screening is positive (low TRECs) or clinical suspicion arises, immediately evaluate lymphocyte subsets and *IL2RG* gene sequence. Once X-SCID is confirmed (or strongly suspected in a boy with T^−^B^+^NK^−^ immunophenotype), move to protective isolation and prophylaxis without delay (35).
2. Stabilization – Initiate IVIG replacement, antibacterial/antifungal/PCP prophylaxis (36), and aggressively treat any existing infections. Do not give live vaccines. Engage an immunology–transplant team early.
3. Donor Search – Start HLA typing family for an MSD; simultaneously, search international donor registries for a MUD. If no immediate match, plan for haploidentical parental donor (commonly the father, to avoid maternal T cells that the baby may harbor).
4. Definitive Therapy Decision – If a matched sibling is available, proceed to urgent transplant (often without or with minimal conditioning, depending on center protocol). If matched unrelated donor is found quickly, that is also an option (with RIC). If neither, move forward with haploidentical transplant (typically T-cell depleted ex-vivo or *in-vivo* with PT-CY) – currently many centers will use low-dose conditioning even for haploidentical donors, given improved immune reconstitution outcomes (43)).
5. Gene Therapy Option – If the patient is identified in a center with a gene therapy trial (and especially if no suitable donor), consider enrolling the patient for gene therapy. For infants, gene therapy may be preferable to a mismatched transplant if trial access is possible. For older patients with partial phenotype, gene therapy can be a rescue if prior transplant failed or was not done.
6. Transplant/Gene Therapy – Carry out the HSCT or gene-corrected cell infusion. Provide supportive care through the immune reconstitution period (sterile isolation, prophylaxis, nutritional support). Monitor for engraftment (chimerism studies) and complications (GVHD in HSCT, or clonal expansions associated with insertional mutagenesis in gene therapy).
7. Post-treatment monitoring – After immune reconstitution, track T-cell counts, T-cell function (proliferation assays), B-cell recovery (check immunoglobulin levels and vaccine titers off IVIG), and chimerism. Continue IVIG until the patient demonstrates robust antibody production and vaccine response (43). Continue prophylaxis (especially PCP prophylaxis) until T cells are clearly adequate (often 6–12 months post-transplant).
8. Long-term care – Vaccinate the patient (all inactivated vaccines once immune reconstitution is sufficient, and eventually live vaccines if T-cell function normalizes – typically after 2 years and off immunosuppression). Monitor for late effects: endocrine (thyroid, fertility), growth, pulmonary function (especially if chronic lung damage from pre-transplant infections), dental issues, neurological and neurodevelopmental problems, and malignancy surveillance. Educate the family that the child’s immune system is essentially restored, but periodic check-ins with immunology are needed.

By following such an algorithm, survival for X-SCID has improved dramatically. It is emphasized that early intervention is lifesaving – in the modern era, no child with X-SCID should die from the condition, given the tools of screening, transplant, and gene therapy. Even patients diagnosed late or those with atypical IL2RG deficiency (who might have been managed as CVID) should be evaluated for curative therapy if they have significant immune dysfunction.

## Conclusion and future directions

IL2RG-related immunodeficiencies exemplify how a single gene defect can disrupt multiple immune pathways, yielding a broad clinical spectrum. Over the last decade, we have gained a much deeper understanding of the γ_c_ cytokine signaling network and how its impairment leads to immunodeficiency. We now appreciate that *IL2RG* mutations are not only a cause of infantile SCID but can also underlie “hidden” immunodeficiencies presenting later in life with atypical features such as HLH, chronic viral infections, granulomatous lesions or antibody failure. This evolving knowledge has practical implications: clinicians should consider genetic testing for *IL2RG* defects in any male patient with unexplained combined immunodeficiency or atypical infections, even outside of infancy. From a mechanistic standpoint, recent studies have illuminated the distinct roles of each γ_c_ cytokine in shaping immune development – reaffirming IL-7 as the driver of T lymphopoiesis (7), IL-15 as indispensable for NK cells (1), and IL-21 as critical for terminal B cell differentiation (41, 42). These insights reinforce why IL2RG is such a critical non-redundant gene.

Therapeutically, the past decade has been transformative. Newborn screening for SCID has been widely implemented (all 50 US states and many other countries worldwide), enabling pre-symptomatic diagnosis of IL2RG SCID and timely treatment (38). As a result, most infants with X-SCID are now transplanted successfully before infections strike, leading to improved overall survival, normal growth and development (39). For those with atypical disease missed by screening, increasing awareness and genetic diagnostics offer a second chance at diagnosis later in life. Meanwhile, HSCT outcomes continue to improve – refined conditioning regimens have solved the historical issue of B-cell reconstitution, and multicenter collaborations (like PIDTC and SCETIDE) have standardized protocols to achieve >90% survival in optimal cases (2, 38, 39). Importantly, these survivors are being followed into adulthood, highlighting issues such as HPV susceptibility and late effects that need ongoing management (38, 45). The field is also witnessing the maturation of gene therapy from experimental to potentially standard practice. The initial setbacks of leukemogenesis have been addressed with safer vector designs, and gene therapy for X-SCID has replicated the curative success of transplant without the need for a donor (28). As gene therapy trials expand (including to older patients and international sites) and long-term follow-up confirms safety, we can expect regulatory approvals and broader availability in the near future. Additionally, genome editing offers a tantalizing future avenue – in the coming years, it may be feasible to correct a baby’s IL2RG mutation *ex vivo* and reinfuse their own edited HSCs, permanently curing the disease with precision and minimal off-target effects (43).

From a research perspective, IL2RG deficiency remains a valuable model to study human immunobiology. Continued analysis of hypomorphic cases is teaching us about partial cytokine signaling and immune regulation – for instance, why do some γ_c_ signals compensate when others are absent? Why do certain *IL2RG* mutations permit T-cell development and survival but not full function? Ongoing studies of patients and mouse models will further parse the contributions of individual γ_c_ cytokines to immunity (7). On the translational side, efforts are underway to reduce the toxicity of conditioning (e.g. using antibody-based bone marrow conditioning instead of chemotherapy) (43), which could make both HSCT and gene therapy safer for SCID infants. There is also interest in late effects: as SCID patients live decades post-transplant, careful monitoring is needed for complications like organ dysfunction, neurodevelopmental issues, and secondary malignancies (which can arise from long-standing HPV infection or from previous gene therapy vector insertions, etc.) (38, 43, 45). Fortunately, thus far X-SCID survivors have not shown a higher incidence of malignancy beyond what might be expected from those specific risk factors.

In conclusion, IL2RG-related immunodeficiencies have evolved from uniformly fatal disorders to ones with an excellent prognosis due to early detection and curative treatments. The common gamma chain continues to be a focus of cutting-edge research, from unraveling cytokine signaling intricacies to pioneering gene therapies. Clinicians should remain vigilant for atypical presentations, as timely diagnosis of an *IL2RG* gene defect – even in a teenager or adult – can open the door to curative therapy. The story of IL2RG exemplifies the trajectory of modern immunology: understanding the molecular basis of disease enables rationally designed interventions that transform patient outcomes. With ongoing advances, it is realistic to expect that within the next decades, few or no children will have to die from IL2RG-related immunodeficiency – a triumph of science and medicine built on 30 years of research into the common γ_c_ cytokine receptor.
