# Correction: IL2RG-related immunodeficiencies: from SCID to atypical presentations

**DOI:** 10.3389/fimmu.2026.1839148

**Published:** 2026-04-23

**Authors:** Efrossini Briassouli, Nikolaos Marinakis, Vana Spoulou, Luigi DNotarangelo

**Affiliations:** 1 Second Department of Paediatrics, National and Kapodistrian University of Athens (NKUA), Aglaia Kyriakou Children’s Hospital, Athens, Greece; 2 Laboratory of Genetics, Department of Medicine, Democritus University of Thrace, Alexandroupolis, Greece; 3 First Department of Paediatrics & Immunobiology & Vaccinology Research Lab, National and Kapodistrian University of Athens, “Agia Sophia” Children’s Hospital of Athens, Athens, Greece; 4 Laboratory of Clinical Immunology and Microbiology, National Institute of Allergy and Infectious Diseases (NIAID), National Institutes of Health (NIH), Bethesda, MD, United States

**Keywords:** atypical X-CID, IL2RG, leaky SCID, maternal T-cell engraftment, somatic reversion, X-SCID

There was a mistake in **Figure 1** as published. The published figure did not accurately reflect the intended final schematic overview of common γ-chain (IL2RG/CD132)-dependent cytokine receptor signaling. The corrected **Figure 1** appears below.

**Figure 1:**
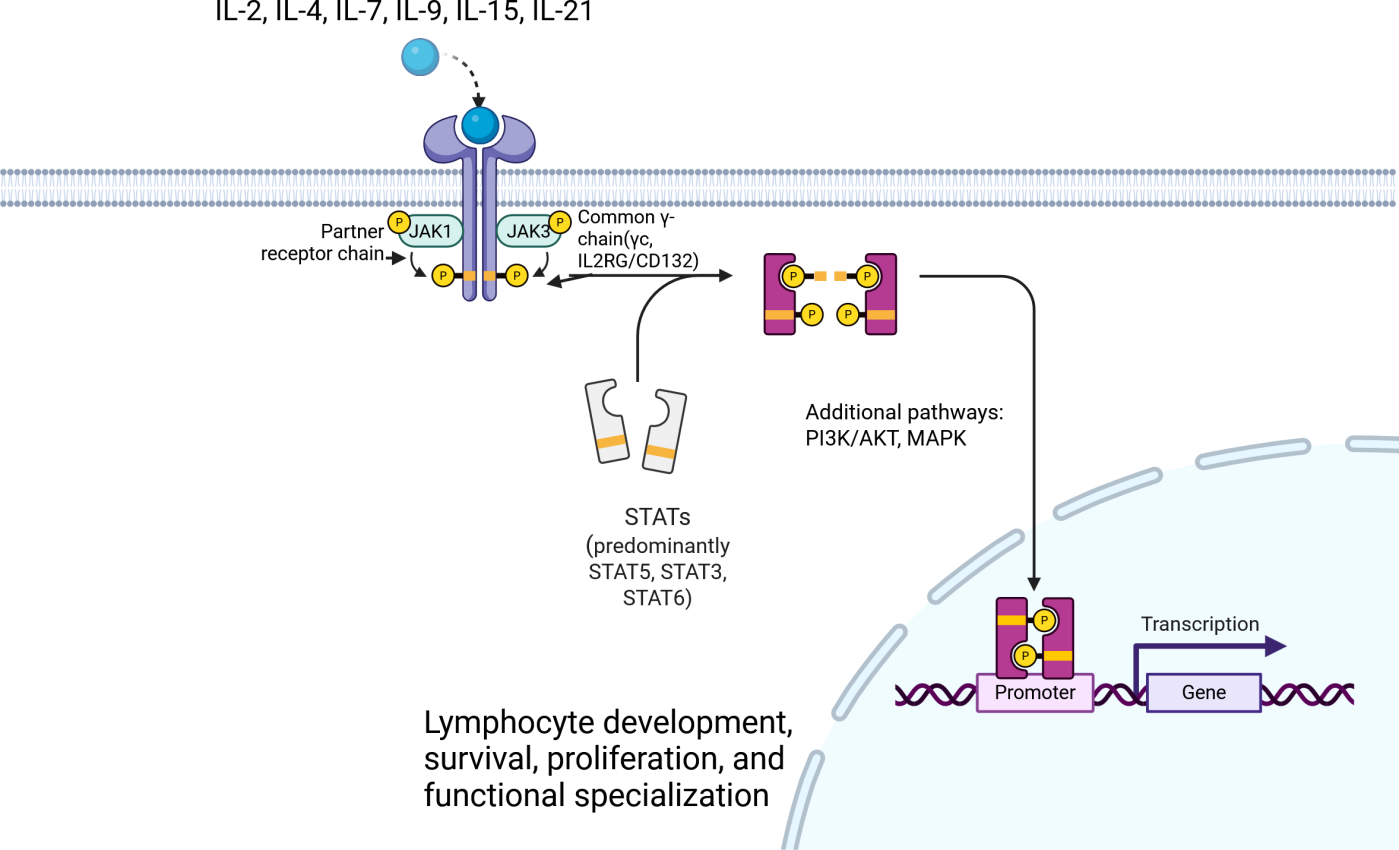
Schematic overview of common γ-chain (IL2RG/CD132)-dependent cytokine receptor signaling. The common γ-chain (γc, IL2RG/CD132) is shared by the receptor complexes for IL-2, IL-4, IL-7, IL-9, IL-15, and IL-21. Cytokine binding promotes receptor assembly with the appropriate partner chains and activation of JAK1 and JAK3, leading predominantly to STAT phosphorylation, dimerization, and nuclear translocation, with additional engagement of PI3K/AKT and MAPK-related pathways. These signaling cascades are essential for lymphocyte development, survival, proliferation, and functional specialization. Created with BioRender.com.

The original version of this article has been updated.

There was a mistake in the caption of **Figure 1** as published. The published caption did not accurately match the corrected schematic. The corrected caption of **Figure 1** appears below.

The original version of this article has been updated.

