# Supplementary material for: IL2RG-related immunodeficiencies: from SCID to atypical presentations
Source: Front Immunol. 2026 Mar 13;17:1703097. doi: 10.3389/fimmu.2026.1703097 (PMC13021486; doi:10.3389/fimmu.2026.1703097)
Supplement: Supplementary file 1 [file Table1.docx]

**Supplementary Table 1.** Curated germline IL2RG variants included in this Review (n=96), listed with HGVS nomenclature (cDNA/protein), dbSNP identifiers, predicted molecular consequence (Sequence Ontology terms), final germline classification, and the ACMG/AMP evidence codes applied. Classifications reflect the evidence available at the time of curation (accessed 25 August 2025).

| **Name** | **dbSNP ID** | **Molecular consequence** | **Germline classification** | **ACMG/AMP criteria** |
| --- | --- | --- | --- | --- |
| c.130_139del (p.Thr44fs) |  | frameshift variant | Pathogenic | PVS1 PM2_Supporting |
| c.147_169dup (p.Leu57fs) | rs2092262555 | frameshift variant | Pathogenic | PVS1 PM2_Supporting |
| c.148del (p.Leu50fs) | rs2147751090 | frameshift variant | Pathogenic | PVS1 PM2_Supporting |
| c.205_215del (p.Tyr69fs) | rs2147750934 | frameshift variant | Pathogenic | PVS1 PM2_Supporting |
| c.207_211dup (p.Asn71delinsThrTer) | rs1556330963 | nonsense | Pathogenic | PVS1 PM2_Supporting |
| c.235_236delinsT (p.Glu79fs) |  | frameshift variant | Likely pathogenic | PVS1 PM2_Supporting |
| c.257C>A (p.Thr86Asn) |  | missense variant | Likely pathogenic | PM1 PM2_Supporting PP3 |
| c.258_261dup (p.His88fs) | rs1556330940 | frameshift variant | Pathogenic | PVS1 PM2_Supporting |
| c.260T>C (p.Leu87Pro) | rs1057520293 | missense variant | Likely pathogenic | PM1 PM2_Supporting PP3 |
| c.268_269del (p.Trp90fs) |  | frameshift variant | Pathogenic | PVS1 PM2_Supporting |
| c.269+1G>A |  | splice donor variant | Pathogenic | PVS1 PM2_Supporting |
| c.270G>A (p.Trp90Ter) | rs1569480047 | nonsense | Pathogenic | PVS1 PM2_Supporting |
| c.302_384dup (p.Val129fs) | rs2147750252 | frameshift variant | Pathogenic | PVS1 PM2_Supporting |
| c.326_340del (p.Glu109_Ser113del) |  | inframe_deletion\|inframe_indel | Likely pathogenic | PM1 PM2_Supporting PM4 |
| c.328del (p.Glu110fs) | rs1064793338 | frameshift variant | Pathogenic | PVS1 PM2_Supporting |
| c.340G>A (p.Gly114Ser) |  | missense variant | Likely pathogenic | PM1 PM2_Supporting PM5 PP3 |
| c.340G>T (p.Gly114Cys) | rs2147750359 | missense variant | Likely pathogenic | PM1 PM2_Supporting PM5 PP3 |
| c.341G>A (p.Gly114Asp) | rs111033620 | missense variant | Pathogenic | PM1 PM2_Supporting PM5 PP3 |
| c.343T>C (p.Cys115Arg) | rs111033622 | missense variant | Pathogenic | PM1 PM2_Supporting PM5 PP3 |
| c.344G>A (p.Cys115Tyr) | rs1556330755 | missense variant | Pathogenic | PM1 PM2_Supporting PM5 PP3 |
| c.344G>T (p.Cys115Phe) | rs1556330755 | missense variant | Pathogenic | PS3 PM1 PM2_Supporting PM5 PP3 |
| c.350_351insA (p.Gln118fs) |  | frameshift variant | Pathogenic | PVS1 PM2_Supporting |
| c.352C>T (p.Gln118Ter) |  | nonsense | Pathogenic | PVS1 PM2_Supporting |
| c.355A>T (p.Lys119Ter) | rs137852507 | nonsense | Pathogenic | PVS1 PM2_Supporting |
| c.359dup (p.Glu121fs) | rs2147750311 | frameshift variant | Pathogenic | PVS1 PM2_Supporting |
| c.365T>A (p.Ile122Asn) | rs2147750291 | missense variant | Likely pathogenic | PM1 PM2_Supporting PP3 |
| c.374A>G (p.Tyr125Cys) | rs2092261313 | missense variant | Likely pathogenic | PM1 PM2_Supporting PP3 |
| c.376del (p.Gln126fs) |  | frameshift variant | Pathogenic | PVS1 PM2_Supporting |
| c.391C>T (p.Gln131Ter) | rs1131691652 | nonsense | Pathogenic | PVS1 PM2_Supporting |
| c.394del (p.Leu132fs) |  | frameshift variant | Pathogenic | PVS1 PM2_Supporting |
| c.427del (p.Thr143fs) | rs1556330710 | frameshift variant | Pathogenic | PVS1 PM2_Supporting |
| c.430C>T (p.Gln144Ter) | rs1602289411 | nonsense | Pathogenic | PVS1 PM2_Supporting |
| c.437T>C (p.Leu146Pro) |  | missense variant | Likely pathogenic | PM1 PM2_Supporting PP3 |
| c.454+1G>A | rs1569480018 | splice donor variant | Pathogenic | PVS1 PM2_Supporting |
| c.455T>G (p.Val152Gly) | rs193922348 | missense variant | Pathogenic | PM1 PM2_Supporting PM5 PP3 |
| c.458T>A (p.Ile153Asn) | rs111033621 | missense variant | Pathogenic | PM1 PM2_Supporting PM5 PP3 |
| c.458T>C (p.Ile153Thr) | rs111033621 | missense variant | Pathogenic | PM1 PM2_Supporting PM5 PP3 |
| c.465G>A (p.Trp155Ter) | rs1569479994 | nonsense | Likely pathogenic | PVS1 PM2_Supporting |
| c.480_481del (p.Leu162fs) |  | frameshift variant | Likely pathogenic | PVS1 PM2_Supporting |
| c.481del (p.Thr161fs) |  | frameshift variant | Likely pathogenic | PVS1 PM2_Supporting |
| c.485T>G (p.Leu162Arg) | rs2092260648 | missense variant | Likely pathogenic | PM1 PM2_Supporting PP3 |
| c.489_490del (p.His163fs) |  | frameshift variant | Likely pathogenic | PVS1 PM2_Supporting |
| c.511G>T (p.Glu171Ter) |  | nonsense | Likely pathogenic | PVS1 PM2_Supporting |
| c.514C>A (p.Leu172Met) | rs141707292 | missense variant | Likely pathogenic | PM1 PM2_Supporting PM5 PP3 |
| c.515T>A (p.Leu172Gln) |  | missense variant | Likely pathogenic | PM1 PM2_Supporting PP3 |
| c.522G>A (p.Trp174Ter) | rs1556330568 | nonsense | Pathogenic | PVS1 PM2_Supporting |
| c.537_538del (p.Asn180fs) |  | frameshift variant | Likely pathogenic | PVS1 PM2_Supporting |
| c.545G>C (p.Cys182Ser) | rs1064794027 | missense variant | Pathogenic | PS4 PM1 PM2_Supporting PM5 PP3 |
| c.546_549del (p.Cys182fs) | rs2147749701 | frameshift variant | Pathogenic | PVS1 PM2_Supporting |
| c.548del (p.Leu183fs) | rs1556330562 | frameshift variant | Pathogenic | PVS1 PM2_Supporting |
| c.550G>T (p.Glu184Ter) | rs2092260520 | nonsense | Pathogenic | PVS1 PM2_Supporting |
| c.551A>G (p.Glu184Gly) |  | missense variant | Likely pathogenic | PM1 PM2_Supporting PP3 |
| c.562C>T (p.Gln188Ter) | rs1556330552 | nonsense | Pathogenic | PVS1 PM2_Supporting |
| c.594+1G>A |  | splice donor variant | Pathogenic | PVS1 PM2_Supporting |
| c.594+2_594+3del | rs1602289183 | splice donor variant | Pathogenic | PVS1 PM2_Supporting |
| c.594+5G>A | rs2147749578 | intron variant | Likely pathogenic | PVS1 PM2_Supporting |
| c.594+5G>T | rs2147749578 | intron variant | Likely pathogenic | PVS1 PM2_Supporting |
| c.598C>T (p.Gln200Ter) | rs2147748363 | nonsense | Pathogenic | PVS1 PM2_Supporting |
| c.602C>G (p.Ser201Ter) | rs1556330286 | nonsense | Pathogenic | PVS1 PM2_Supporting |
| c.603_604del (p.Val202fs) | rs2092258288 | frameshift variant | Pathogenic | PVS1 PM2_Supporting |
| c.612T>A (p.Tyr204Ter) | rs2147748344 | nonsense | Pathogenic | PVS1 PM2_Supporting |
| c.662T>C (p.Phe221Ser) | rs193922349 | missense variant | Likely pathogenic | PM1 PM2_Supporting PP3 |
| c.664C>A (p.Arg222Ser) |  | missense variant | Likely pathogenic | PM1 PM2_Supporting PP3 |
| c.664C>G (p.Arg222Gly) |  | missense variant | Likely pathogenic | PM1 PM2_Supporting PP3 |
| c.667G>T (p.Val223Phe) | rs2092258151 | missense variant | Likely pathogenic | PM1 PM2_Supporting PP3 |
| c.671G>C (p.Arg224Pro) |  | missense variant | Likely pathogenic | PM1 PM2_Supporting PP3 |
| c.675C>A (p.Ser225Arg) | rs1569479913 | missense variant | Pathogenic | PS1 PM1 PM2_Supporting PP3 |
| c.675C>G (p.Ser225Arg) | rs1569479913 | missense variant | Pathogenic | PS1 PM1 PM2_Supporting PP3 |
| c.681del (p.Phe227fs) |  | frameshift variant | Likely pathogenic | PVS1 PM2_Supporting |
| c.692G>A (p.Cys231Tyr) |  | missense variant | Likely pathogenic | PM1 PM2_Supporting PP3 |
| c.695G>T (p.Gly232Val) | rs2147748189 | missense variant | Likely pathogenic | PM1 PM2_Supporting PP3 |
| c.703_711dup (p.Gln235_Trp237dup) | rs587776729 | inframe_insertion | Pathogenic | PM1 PM2_Supporting PM4 |
| c.711G>C (p.Trp237Cys) |  | missense variant | Likely pathogenic | PM1 PM2_Supporting PP3 |
| c.713G>A (p.Ser238Asn) | rs2147748142 | missense variant | Likely pathogenic | PM1 PM2_Supporting PP3 |
| c.715G>T (p.Glu239Ter) |  | nonsense | Likely pathogenic | PVS1 PM2_Supporting |
| c.718T>C (p.Trp240Arg) | rs1057517950 | missense variant | Likely pathogenic | PM1 PM2_Supporting PM5 PP3 |
| c.722_724dup (p.Ser241_His242insArg) |  | inframe_insertion | Likely pathogenic | PM2_Supporting PM4 |
| c.758-1G>C | rs886042051 | splice acceptor variant | Pathogenic | PVS1 PM2_Supporting |
| c.816_819del (p.Ile273fs) | rs2147747375 | frameshift variant | Pathogenic | PVS1 PM2_Supporting |
| c.820_823dup (p.Ser275fs) |  | frameshift variant | Likely pathogenic | PVS1 PM2_Supporting |
| c.829del (p.Leu277fs) |  | frameshift variant | Pathogenic | PVS1 PM2_Supporting |
| c.846G>A (p.Trp282Ter) | rs1556329954 | nonsense | Pathogenic | PVS1 PM2_Supporting |
| c.854+2T>C | rs2147747293 | splice donor variant | Pathogenic | PVS1 PM2_Supporting |
| c.855-1G>A | rs1556329822 | splice acceptor variant | Pathogenic | PVS1 PM2_Supporting |
| c.855-2A>C | rs2147746826 | splice acceptor variant | Pathogenic | PVS1 PM2_Supporting |
| c.865dup (p.Arg289fs) |  | frameshift variant | Pathogenic | PVS1 PM2_Supporting |
| c.878T>A (p.Leu293Gln) | rs137852510 | missense variant | Pathogenic | PS3 PM2_Supporting PP1 PP3 |
| c.879del (p.Lys294fs) |  | frameshift variant | Pathogenic | PVS1 PM2_Supporting |
| c.905_906del (p.Glu302fs) | rs2147746709 | frameshift variant | Pathogenic | PVS1 PM2_Supporting |
| c.922del (p.Ser308fs) | rs2147746685 | frameshift variant | Pathogenic | PVS1 PM2_Supporting |
| c.923C>A (p.Ser308Ter) | rs137852509 | nonsense | Pathogenic | PVS1 PM2_Supporting |
| c.924G>C (p.Ser308=) |  | synonymous variant | Pathogenic | PVS1 PM2_Supporting |
| c.943_962del (p.Lys315fs) |  | frameshift variant | Pathogenic | PVS1 PM2_Supporting |
| c.958_959del (p.Leu321fs) | rs1602288051 | frameshift variant | Pathogenic | PVS1 PM2_Supporting |
| c.967_970dup (p.Asp324fs) |  | frameshift variant | Likely pathogenic | PVS1 PM2_Supporting |
| c.980A>G (p.Glu327Gly) | rs1064794631 | missense variant | Likely pathogenic | PP3 PM2_Supporting |
